# Second messenger Ap_4_A polymerizes target protein HINT1 to transduce signals in FcεRI-activated mast cells

**DOI:** 10.1038/s41467-019-12710-8

**Published:** 2019-10-11

**Authors:** Jing Yu, Zaizhou Liu, Yuanyuan Liang, Feng Luo, Jie Zhang, Cuiping Tian, Alex Motzik, Mengmeng Zheng, Jingwu Kang, Guisheng Zhong, Cong Liu, Pengfei Fang, Min Guo, Ehud Razin, Jing Wang

**Affiliations:** 1 State Key Laboratory of Bioorganic and Natural Products Chemistry, Center for Excellence in Molecular Synthesis, Shanghai Institute of Organic Chemistry, University of Chinese Academy of Sciences, Chinese Academy of Sciences, 345 Lingling Road, Shanghai, 200032 China; 2 Interdisciplinary Research Center on Biology and Chemistry, Shanghai Institute of Organic Chemistry, Chinese Academy of Sciences, Shanghai, 201210 China; 3 iHuman Institute, ShanghaiTech University, Shanghai, 201210 China; 4 Department of Biochemistry and Molecular Biology, Institute for Medical Research Israel-Canada, Hebrew University Medical School, Jerusalem, 91120 Israel; 5 Kangma BioTech, Co., Ltd, 1131 Cailun Road, Shanghai, 201203 China; 6 NUS-HUJ-CREATE Cellular and Molecular Mechanisms of Inflammation Program, Department of Microbiology and Immunology, National University of Singapore, Singapore, 117597 Singapore

**Keywords:** Stress signalling, Transcriptional regulatory elements, X-ray crystallography

## Abstract

Signal transduction systems enable organisms to monitor their external environments and accordingly adjust the cellular processes. In mast cells, the second messenger Ap_4_A binds to the histidine triad nucleotide-binding protein 1 (HINT1), disrupts its interaction with the microphthalmia-associated transcription factor (MITF), and eventually activates the transcription of genes downstream of MITF in response to immunostimulation. How the HINT1 protein recognizes and is regulated by Ap_4_A remain unclear. Here, using eight crystal structures, biochemical experiments, negative stain electron microscopy, and cellular experiments, we report that Ap_4_A specifically polymerizes HINT1 in solution and in activated rat basophilic leukemia cells. The polymerization interface overlaps with the area on HINT1 for MITF interaction, suggesting a possible competitive mechanism to release MITF for transcriptional activation. The mechanism depends precisely on the length of the phosphodiester linkage of Ap_4_A. These results highlight a direct polymerization signaling mechanism by the second messenger.

## Abstract (web summary)

The second messenger Ap_4_A contributes to mast cell activation by interacting with HINT1 but the molecular mechanism is not fully understood. Here, the authors show that Ap_4_A regulates downstream signaling by polymerizing HINT1 and provide insights into how specificity over other second messengers is achieved.

## Introduction

Second messengers propagate extracellular signals that are initiated by the binding of first messengers (e.g., hormones, allergens, and neurotransmitters) to cell membrane-bound receptors. By binding and activating target proteins including kinases, ion channels, or regulatory proteins, second messengers relay signaling cascades in the cytoplasm or nucleus^1–4^. Diadenosine-5′,5-P1,P4-tetraphosphate (Ap_4_A) is a ubiquitous second messenger from bacteria to higher eukaryotes^5,6^, which responds to environmental stresses and cell cycle phases^7–14^.

Mast cells, the principle effector for all allergic responses^15,16^, depend on Ap_4_A for cell signaling^14,17–19^. IgE, together with antigen stimulation, triggers the phosphorylation of cytoplasmic lysyl-tRNA synthetase (LysRS) on the S207 residue through the mitogen-activated protein kinase (MAPK) pathway^18^. The phosphorylated LysRS no longer functions in translation, but gains the ability to synthesize Ap_4_A molecules^14,18–20^. Consequently, cellular Ap_4_A levels accumulate up to ~775 µM within 15 min in bone marrow-derived mast cells (BMMCs)^14,17^. Ap_4_A then binds to the histidine triad nucleotide-binding protein 1 (HINT1), thereby disrupting the protein–protein interaction with microphthalmia-associated transcription factor (MITF), and the released MITF eventually promotes the transcription of mast cell activation genes^14,18,20,21^. Inhibition of the MAPK kinases or knockdown of LysRS by siRNA markedly reduces the intracellular level of Ap_4_A and suppresses the transcription of MITF target genes^18^. The restoration from the stimulated response is mediated by the Ap_4_A hydrolase^17^. Knocking down the Ap_4_A hydrolase in rat basophilic leukemia (RBL) cells, which is widely used in allergy studies as a mast cell model^22^, leads to the accumulation of cellular Ap_4_A, prolonged dissociation of MITF from HINT1, and increased transcription of MITF target genes^17^.

MITF is a key transcription factor that regulates the expression of many genes critical for mast cell activation including proteases, cytokine receptors, and cell adhesion molecules^23–26^. Mice lacking a functional *Mitf* gene (*mi*/*mi*) are essentially deficient in mast cells^23,27^ and are susceptible to death from parasite infections^28^. Co-transfection of MITF with HINT1 inhibited up to 94% of the MITF-mediated transcriptional activation of mast cell protease-6, which indicates that HINT1 is an important regulator of MITF during mast cell activation^21^. Besides the MAPK-LysRS-Ap_4_A-HINT1 signaling pathway, multiple pathways have been reported to regulate MITF activity and/or stability in mast cells, including the c-KIT and PI3K pathways^29,30^.

HINT1 knockout mice (*Hint1*^−/−^) showed hyperalgesia caused by allergic or inflammatory responses, indicating aberrant mast cell activation^31^. Both *Hint1*^−/−^ and *Hint1*^−/+^ mice had high incidences of tumorigenesis after the exposure to the carcinogens 7,12-dimethylbenz(a)anthracene or *N*-nitrosomethylbenzylamine^32,33^. These phenotypes are consistent with the postulation that activated mast cells infiltrate tumors, thereby releasing proangiogenic factors that accelerate malignant progression^34^.

However, how HINT1 is regulated by Ap_4_A remains unclear. Here we report that Ap_4_A specifically polymerizes HINT1 into (HINT1-Ap_4_A)_*n*_ (HAn) filaments in solution and in the activated RBL cells. HINT1 polymerization blocks the potential MITF interaction interface, suggesting a competitive mechanism that releases MITF and leads to transcriptional activation in response to the IgE-FcεRI stimulation. This mechanism depends precisely on the length of the phosphodiester linkage of Ap_4_A, explaining why other di-adenosine polyphosphates (Ap_*n*_A) family members such as di-adenosine triphosphate (Ap_3_A) or di-adenosine pentaphosphate (Ap_5_A) do not mediate this pathway^14^. Together, these results highlight a direct polymerization-mediated signaling mechanism for the second messenger Ap_4_A in the human innate immune response.

## Results

### Ap_4_A integrated two HINT1 dimers into a tetramer in crystal

**Fig. 1:**
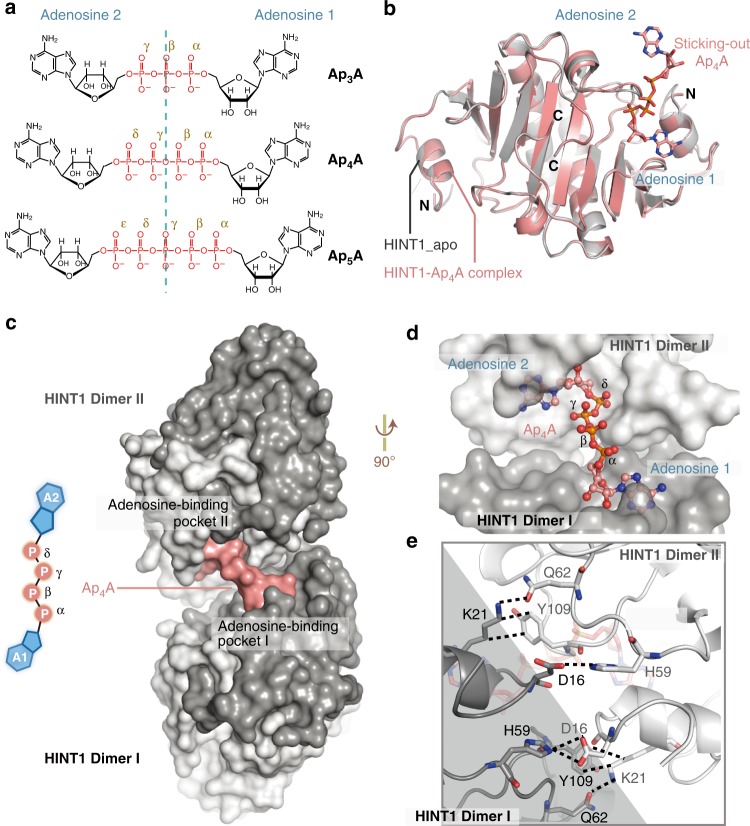
Ap_4_A integrated two HINT1 dimers into a HINT1-Ap_4_A-HINT1 structure. **a** Schematics of adenosine polyphosphates (Ap_*n*_A). Ap_*n*_A is composed by two adenosine moieties linked through ribose 5′-carbons to a phosphate group chain of different length (*n* denotes the number of phosphate groups). **b** Superimposition of the HINT1-Ap_4_A complex structure (pink) and HINT1_apo structure (PDB: 1KPB, gray). **c** Two HINT1 dimers (Dimer I and Dimer II) were integrated into a tetramer in the HINT1-Ap_4_A complex structure. HINT1 proteins are shown as surface in black or white, and the Ap_4_A molecule is shown as pink surface. **d** A new cleft built by two adenosine pockets with a long groove accommodated the Ap_4_A molecule. The HINT1 proteins are shown as surface and the Ap_4_A molecule is shown as sticks. **e** Zoom-in view of the Ap_4_A-induced tetramer interface between HINT1 Dimer I (black) and Dimer II (white). The interacting residues are shown as sticks

To understand how Ap_4_A regulates HINT1, we set out to determine the crystal structure of human HINT1 in complex with Ap_4_A. HINT1 was reported to efficiently hydrolyze aminoacyl-adenylate (aa-AMP) and ADP, but neither ATP nor Ap_*n*_A (such as Ap_3_A, Ap_4_A, and Ap_5_A; Fig. 1a)^35–37^. Incubation of 1 mM Ap_4_A with 25 μM HINT1 at 37 °C for 24 h showed minimal (<5%) hydrolysis (Supplementary Fig. 1). Therefore, we started with wild-type (WT) HINT1 to co-crystallize with Ap_4_A and solved the HINT1_WT_-Ap_4_A structure at a resolution of 1.52 Å. For comparison, we also solved the HINT1_WT_-ATP and HINT1_WT_-Ap_5_A structures at resolutions of 1.52 and 1.31 Å, respectively (Supplementary Table 1). Consistent with previous reports^35,37^, two HINT1 molecules formed a dimer through the five-stranded β-sheet and the central helix in the crystal structure (Supplementary Fig. 2a). Despite having co-crystallized with different ligands, the three structures were nearly identical, with root mean square deviations (RMSDs) < 0.083 Å (Supplementary Fig. 2a). However, in all the three structures, only the electron density corresponding to an AMP molecule was observed in the adenosine pocket (Supplementary Fig. 2b, c), indicating that the ligands had been hydrolyzed by the HINT1_WT_ protein during the crystallization, which took 3–7 days at 18 °C with a high protein concentration of ~2 mM.

We then made an H114A mutant to inactivate the hydrolysis activity of HINT1^37^. The complex structures of HINT1_H114A_ with Ap_4_A were then obtained through both co-crystallization (HINT1_H114A_-Ap_4_A^cocrystallization^, 0.95 Å) and soaking methods (HINT1_H114A_-Ap_4_A^soaking^, 1.42 Å) (Supplementary Table 1). The complete Ap_4_A molecule was observed in one adenosine pocket of a HINT dimer (denoted as HINT1 Dimer I) in both co-crystallization and soaking structures (Fig. 1b and Supplementary Fig. 3). The other adenosine pocket of the same HINT1 dimer is vacant of Ap_4_A, because it is blocked by the crystal packing (Supplementary Fig. 3a, b). In both structures, one adenosine (A1) and the α-phosphate (Pα) of Ap_4_A bind inside the pocket (Fig. 1b and Supplementary Fig. 4a), whereas the rest of the molecule adopts a unique Sticking-out conformation above the HINT1 surface (Fig. 1b). This conformation is significantly different from the flat conformation of Ap_3_A in the pocket of the homologous fragile histidine triad protein^38^, due to the distinct C-terminal structures of the proteins (Supplementary Fig. 5). Interestingly, the Ap_4_A-complexed structure closely resembled the HINT1 apo structure with an overall RMSD about 0.238 Å (Fig. 1b and Supplementary Fig. 4b). Only three positions including the residues S107, I32, and D16 surrounding the adenosine pocket had minor shifts of 1.3, 3.8, and 1.9 Å upon Ap_4_A binding (Supplementary Fig. 4b–e). This was distinct from the large conformational change of target proteins induced by second messengers in the previous studies^39–46^. These results suggested that Ap_4_A might regulate HINT1 through a distinct mechanism rather than inducing large, global conformational changes.

Of note, the sticking-out portion of Ap_4_A, containing the second adenosine (A2) and the delta phosphate (Pδ), fit into an adjacent HINT1 dimer in the crystal structure (denoted as HINT1 Dimer II; Fig. 1c and Supplementary Fig. 3). The two HINT1 dimers formed a large cleft with their adjoining adenosine pockets that accommodated Ap_4_A (Fig. 1c, d). The HINT1 dimers I and II not only bound the same Ap_4_A molecule but also interacted with each other directly. The interacting residues include D16, I18, K21, H59, Q62, S107, and Y109, which is denoted as Ap_4_A-linked interface in Fig. 1e. Therefore, in the presence of Ap_4_A, two HINT1 dimers were integrated into a compact (HINT1)_2_-Ap_4_A-(HINT1)_2_ tetramer in the crystalline state.

The human HINT1 protein was previously co-crystallized with an Ap_4_A analog JB419^47^. In the JB419 molecule, the phosphate linkage was replaced by a non-hydrolysable bis-phosphorothioated glycerol structure (Supplementary Fig. 6a). Consistently, JB419 was shown to tether two HINT1 dimers into a tetramer in the crystal, supporting our observation that the two symmetric adenosine moieties could lead to HINT1 tetramerization (Supplementary Fig. 6b).

Interestingly, the relative orientation and detailed tetramer interface of HINT1 linked by JB419 and Ap_4_A are quite different (rotated ~44°; Supplementary Fig. 6b). The resulting tetramer interface induced by JB419 is composed of I18, I44, S45, S102, S107, and Q120, which is significantly different from the tetramer interface in the HINT1-Ap_4_A-HINT1 structure, which is composed by D16, I18, K21, H59, Q62, S107, and Y109 (Supplementary Fig. 6d, e). These differences further expand to the flexible linker in JB419 compared with the relatively rigid and negative-charged phosphate linker of Ap_4_A that is involved in the HINT1-Ap_4_A interaction (Supplementary Fig. 6c). Structural analysis shows that the HINT1-JB419-HINT1 tetramer interface does not overlap with the HINT1-MITF interface composed of K21 and Y109, nor the Ap_4_A-linked interface (composed of D16, I18, K21, H59, Q62, S107, and Y109) (Supplementary Fig. 6e). These differences suggest that both the symmetric adenosines of Ap_4_A and its natural tetraphosphate linkage are critical to modulate the orientation of HINT1 and subsequent polymerization.

### Ap_4_A induces HINT1 polymerization in solution

**Fig. 2:**
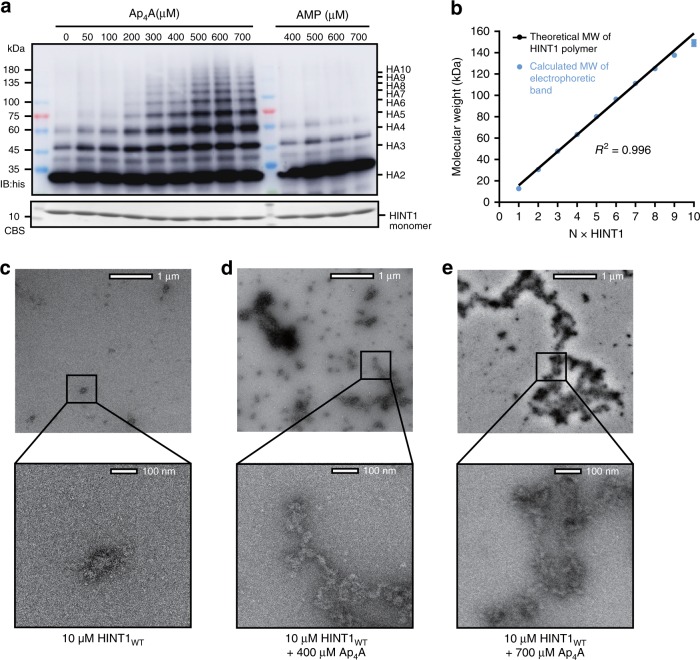
Ap_4_A-promoted HINT1 polymerization. **a** Electrophoretic mobility shift assay (EMSA) was performed with 25 μM HINT1_H114A_ incubated with 0–700 μM Ap_4_A or 400–700 μM AMP. The monomer band of HINT1 was detected by Coomassie brilliant blue staining (CBS) and the polymer bands were detected by western blotting against the 6×his-tag fused with HINT1. The experiment was performed three times with similar results. Source data are provided as a Source Data file. **b** The molecular weights of the experimental polymers correlated with the integral multiples of the theoretical molecular weight of HINT1 with R-squared = 0.996 (error bars represent the SEM of three experimental repeats). Source data are provided as a Source Data file. **c**–**e** Negative stain EM images of HINT1_WT_ without Ap_4_A **c**, in the presence of 400 μM Ap_4_A **d**, or 700 μM Ap_4_A **e**

Crystal packing blocked the other two distal adenosine pockets of the (HINT1)_2_-Ap_4_A-(HINT1)_2_ tetramer (Supplementary Fig. 3a–c). However, in solution, HINT1s with these available pockets should be able to bind additional Ap_4_As to allow polymerization. To confirm the polymer in solution, the HINT1_H114A_ protein was incubated with gradient concentrations of Ap_4_A, from 0 to 700 μM; equivalent to the physiological concentration of Ap_4_A in the activated BMMC cells^14^. HINT1 polymers were monitored by an electrophoretic mobility shift assay (EMSA). As the native Ap_4_A-HINT1 polymers were too large to enter the polyacrylamide gel, we partially destabilized the polymer using sodium dodecyl sulfate (SDS) without heat denaturation. The remaining Ap_4_A-HINT1 oligomer fragments were observed with a sensitive western blotting. Our results show that Ap_4_A induced HINT1 polymerization HAn, resulting in complexes from 31.7 kDa (*n* = 2) to over 142.9 kDa (*n* = 10), in an Ap_4_A dose-dependent manner (Fig. 2a). As SDS breaks both the Ap_4_A-linked interactions and the classic dimer interfaces indiscriminately, polymers with odd numbers of HINT1 were also observed. The molecular weights of the polymers matched the integral multiples of the theoretical molecular weight of HINT1 with an R-squared (*r*^2^) of 0.996 (Fig. 2b). In addition, greater percentages of HINT1 polymers were observed with prolonged incubation in Ap_4_A, with polymerization at 37 °C being more robust than that at 4 °C (Supplementary Fig. 7). By contrast, AMP did not drive HINT1 polymerization (Fig. 2a), indicating that polymerization of HINT1 was specifically induced by the bivalent Ap_4_A molecule.

We further performed analytical ultracentrifuge experiments to quantify HINT1 polymerization. After incubation with Ap_4_A, high-order HINT1-Ap_4_A polymers were observed with the sedimentation coefficient (S) at 45S (~2.2 MDa) and 62S (~4.2 MDa) (Supplementary Fig. 8). Approximately 15% of HINT1 formed high-order polymers after Ap_4_A incubation in this assay. The partial polymerization supported the dynamic nature of the HINT1-Ap_4_A complex during immune activation. To further visualize the Ap_4_A-induced HINT1 polymer in native states, we analyzed the HINT1 samples through negative stain electron microscopy (EM). Incubation with 400 μM Ap_4_A significantly promoted the formation of filament structures in the HINT1 sample (Fig. 2c, d). Of note, single-molecule fibers could not be seen at this resolution; only bundles of single-molecule fibers were observed as filaments. Increasing the Ap_4_A concentration to 700 μM further enhanced the intensity and size of HINT1_WT_ filaments to a length of ~10 µm (10–250 nm in width), roughly the size of ~15,000 HINT1 dimers (Fig. 2e). These observations indicate that Ap_4_A in solution promotes HINT1 to form HAn polymers (*n* from 2 to over 10 K).

### HAn formation breaks HINT1-MITF interactions in solution

**Fig. 3:**
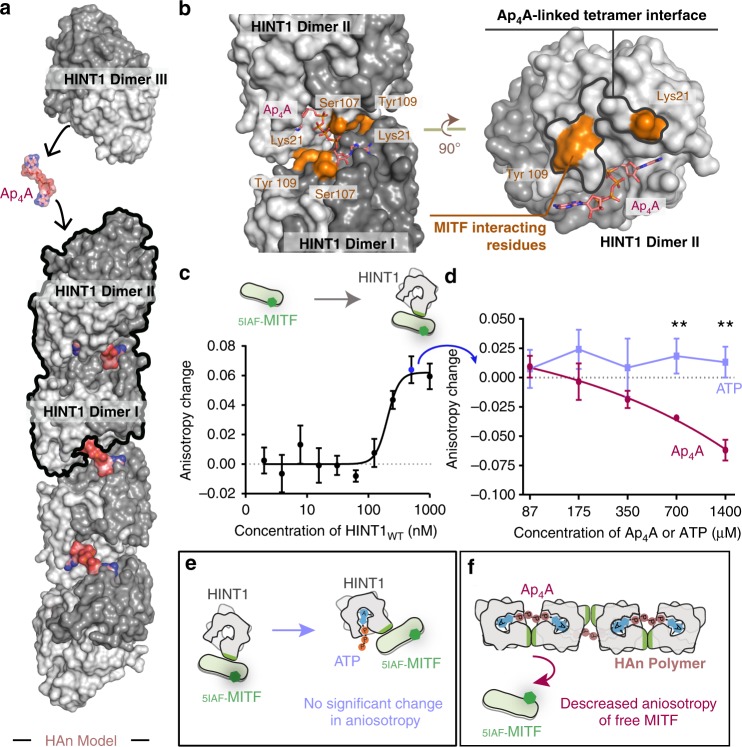
HAn formation breaks HINT1-MITF interactions in solution. **a** A model of (HINT1-Ap_4_A)_*n*_ polymer, denoted as HAn, is built based on the HINT1-Ap_4_A tetramer observed in the crystal structure (circled with a black line). **b** Interface for MITF (labeled as yellow) overlaps with Ap_4_A-induced tetramer interface (circled with a black line). Posttranslational modification sites of HINT1 (yellow) is located at the tetramer interface of HINT1. Among them, Lys21 and Tyr109 were reported to directly bind MITF, and modification on these sites disrupted MITF-HINT1 interaction and released MITF transcription activity. **c** Fluorescence anisotropy assay designed to monitor the interaction between HINT1_WT_ and _5IAF-_MITF. Error bars represent the SD of three experimental repeats. Source data are provided as a Source Data file. **d** Titration of Ap_4_A and ATP into the _5IAF-_MITF-HINT1_WT_ complex at the platform of **c**. Error bars represent the SD of three experimental repeats. **P < 0.01, from two-tailed Student’s *t*-test. Source data are provided as a Source Data file. **e** ATP could not induce HINT1 polymerization and could not release _5IAF-_MITF to decrease the fluorescence anisotropy. **f** Ap_4_A was able to induce HINT1 polymerization and release _5IAF-_MITF to decrease the fluorescence anisotropy

Based on the HINT1-Ap_4_A tetramer crystal structure, a model of HAn polymers can be established. The HINT1 protein is a compact and symmetric dimer, with two adenosine pockets at opposite sides. The dimers I and II formed a tetramer with a shared Ap_4_A bound to their approximated adenosine pockets. The other adenosine pocket of dimer II could interact with an additional Ap_4_A and promote the interaction with a HINT1 dimer III, and so forth to polymerize into a filament (Fig. 3a and Supplementary Fig. 9a). The monomeric mutation V97D on the classic HINT1 dimer interface^48^ significantly reduced HAn formation (Supplementary Fig. 9b, c). Ap_4_A-linked interface mutations K21D and Y109D also disrupted HAn formation (Supplementary Fig. 9d, e). Therefore, both the classic dimer interface and the Ap_4_A-linked interface were essential for HAn formation.

Notably, the Ap_4_A-linked interface on HINT1 is also a hot spot for posttranslational modifications^49^. The acetylation of K21 and phosphorylation of Y109 disrupt the HINT1-MITF interaction, thereby activating MITF transcriptional activity^49^. The extensive overlap between the Ap_4_A-linked interface and the MITF regulation site suggests that the formation of HAn would block the interaction between HINT and MITF (Fig. 3b).

To verify the impact of HAn formation on the MITF-HINT1 interaction, we designed a fluorescence anisotropy assay (Fig. 3c). MITF was labeled with the fluorophore 5IAF (denoted as _5IAF-_MITF). Formation of the HINT1-MITF complex increased the fluorescence anisotropy of _5IAF-_MITF (Fig. 3c). Next, Ap_4_A was titrated into the _5IAF-_MITF-HINT1 complex. As a result, the fluorescent polarization of _5IAF_-MITF decreased in an Ap_4_A dose-dependent manner (Fig. 3d, f), in correlation with the HAn formation induced by Ap_4_A (Fig. 2). By contrast, ATP was not able to break the MITF-HINT1 interaction in this assay (Fig. 3d, e), which again indicates that the HAn effect on MITF was specific to the linkage of Ap_4_A.

### HINT1 polymerizes in stimulated RBL cells

**Fig. 4:**
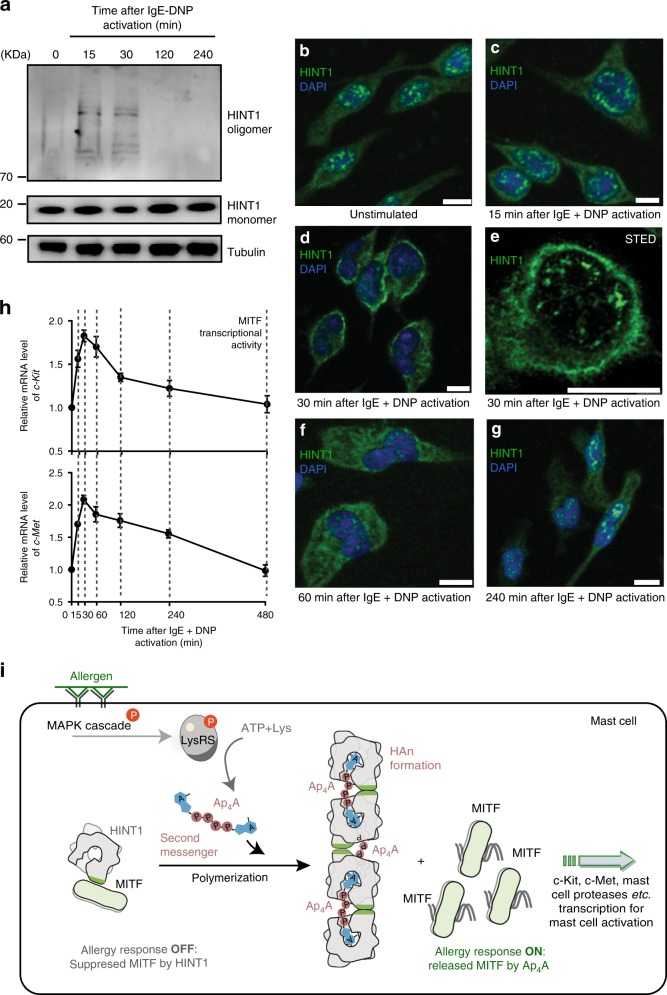
HINT1 polymerizes in stimulated RBL cells. **a** Western blottings of the endogenous HINT1 following by the IgE and antigen stimulation. 0 indicates the unstimulated state. Source data are provided as a Source Data file. **b**–**g** Endogenous HINT1 in Rat basophilic leukemia (RBL) cell following the IgE and antigen stimulation (0–4 h) were immunostained and visualized by confocal laser scanning microscope or stimulated emission depletion microscope (STED). Scale bars, 10 μm. Nuclei were labeled with DAPI. One representative experiment out of three is shown. **h** The transcript level of *c-Met* and *c-Kit* following the IgE and antigen stimulation. Error bars represent the SEM of three experimental repeats. Source data are provided as a Source Data file. **i** Schematic cartoon showing Ap_4_A induces the formation of HAn polymer to release MITF for transcriptional activity in allergic response

Cellular Ap_4_A levels are significantly increased upon mast cell activation with a peak concentration of >180 μM in the activated RBL cells and >700 μM in BMMCs^14^. These concentration fluxes occur within 15–30 min and drop back to the basal level after 1 h^17^. These concentrations are sufficient to induce the formation of HAn in solution (Fig. 2a). Consistently, we observed the polymerization of endogenous HINT1 in activated RBL cells (Fig. 4a). We show that HINT1 forms polymers upon IgE-Antigen stimulation within 15–30 min and decreases after 2 h (Fig. 4a), which was synchronous with the changes in Ap_4_A concentration in the literature^17^.

To further confirm our HAn structure model, we established HINT1 knockdown RBL cells (Supplementary Fig. 10a) and then stably expressed Flag_HINT1_WT and mutants (Y109D- at the Ap_4_A linked interface, could not form polymers in vitro; L53R, I22R at the adenosine binding pocket; V97D at the classic HINT1 dimer interface) in these cells (Supplementary Figs. 9 and 10b). However, only the Y109D mutant was expressed at a similar level as WT protein (Supplementary Fig. 10b) and therefore we focused on the Y109D mutant for further analysis. Using anti-Flag antibody, we observed strong polymers of Flag_HINT1_WT after stimulation (Supplementary Fig. 10c). This phenotype was not seen with the Ap_4_A linked interface mutant Flag_HINT1_Y109D (Supplementary Fig. 10c), which is consistent with our in vitro results (Supplementary Fig. 9e) and supports our hypothesis that HINT1 polymerizes through the proposed HAn structure model in activated RBL cells.

As HINT1 polymerized after stimulation and depolymerized after recovery in the RBL cells (Fig. 4a and Supplementary Fig. 10c), we expected that the cellular distribution of HINT1 would also change during this process. We observed the distribution pattern of the protein using laser confocal microscopy. In the unstimulated RBL cells, HINT1 was present in both the nucleus and cytoplasm. And in the nucleus, it was mostly observed as bright dots (Fig. 4b). Interestingly, punctate localization of MITF in the nucleus has also been reported^21^. We further found that HINT1 and MITF were co-localized on these dots in the unstimulated RBL cells (Supplementary Fig. 11). Fifteen to 30 min after RBL stimulation, the bright HINT1 puncta reduced in the nucleus and the distribution pattern changed obviously (Fig. 4c–e). Four hours after the stimulation, bright HINT1 dots re-formed in the nucleus in a pattern similar to the previously unstimulated state (Fig. 4f, g). Therefore, the changes in distribution pattern of HINT1 were also synchronous with the reported changes in Ap_4_A concentration during the RBL activation.

These data were also consistent with the polymerization of HINT1 and the increased transcription of MITF downstream genes (*c-Met*, *c-Kit*), which reached peak levels at 30 min (Fig. 4h). After the peak, the levels of the *c-Met* and *c-Kit* transcripts declined slowly, and returned to basal level 4–8 h after the stimulation (Fig. 4h). We also determined the mRNA levels of *c-Kit* and *c-Met* in the HINT1 knockdown RBL cells expressing Flag_HINT1_WT and Flag_HINT1_Y109D (Supplementary Fig. 10d). The expression of Flag_HINT1_WT suppressed the mRNA levels of *c-Kit* and *c-Met* in the HINT1 knockdown RBL cells, and recovered the response to the stimulation (Supplementary Fig. 10d). On the contrary, the expression of Flag_HINT1_Y109D, which could not polymerize nor bind MITF, showed increased mRNA levels of *c-Kit* and *c-Met* throughout the stimulation process. In summary, the changes in Ap_4_A concentration, HAn formation, and gene transcription were synchronized in the mast cell activation process.

### Function of Ap_*n*_A is determined by the phosphodiester linkage

**Fig. 5:**
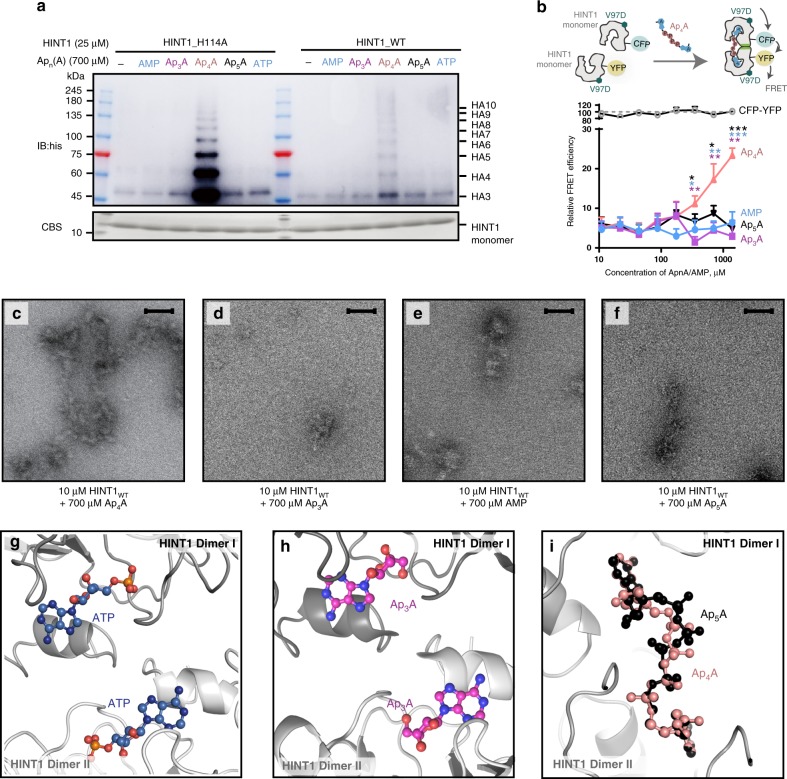
The length-dependent regulation of Ap_4_A on HAn formation. **a** EMSA was performed with 25 μM HINT1_H114A_ or HINT1_WT_ incubated with 700 μM AMP, Ap_3_A, Ap_4_A, Ap_5_A, and ATP separately. Only Ap_4_A could induce the HINT1 polymerization. The experiment was performed three times with similar results. Source data are provided as a Source Data file. **b** FRET assay showing that only Ap_4_A, but not Ap_5_A/Ap_3_A/AMP, could induce the interaction between HINT1_V97D_-CFP and HINT1_V97D_-YFP. FRET signal between CFP-YFP tandems was measured and set to 100% (error bars represent the SD of three experimental repeats). *P < 0.05, **P < 0.01, ***P < 0.001, from two-tailed Student’s *t*-test. Source data are provided as a Source Data file. **c**–**f** Negative stain EM of 10 μM HINT1_WT_ incubated with Ap_4_A, Ap_3_A, AMP, and Ap_5_A separately. Only Ap_4_A could efficiently induce the HINT1 filament formation. Scale bars, 100 nm. **g** The conformation of ATP in the HINT1_H114A_-ATP complex structure. Only AMP portion was visible, suggesting the rest part was flexible or disordered. **h** The conformation of Ap_3_A in the HINT1_H114A_-Ap_3_A complex structure. Only adenosine moiety was visible. **i** The conformation of Ap_5_A in the HINT1_H114A_-Ap_5_A complex structure. Two adenosines of Ap_5_A fit in the two adenosine pockets in HINT1 dimer I and HINT1 dimer II separately. The five-phosphate linkage in Ap_5_A adopted a bent and zigzag conformation, comparing with the straight and tight Ap_4_A

Early studies showed that only Ap_4_A among the Ap_*n*_A family could disrupt the HINT1-MITF interaction and activate MITF^14^. To understand the specificity of Ap_4_A on HINT1 regulation, we investigated the ability of Ap_3_A and Ap_5_A to induce HINT1 polymerization through EMSA. Our results show that Ap_3_A, Ap_5_A, AMP, and ATP could not induce HINT1 polymerization (Fig. 5a). We designed an additional fluorescence resonance energy transfer (FRET) assay based on the monomeric CFP-HINT1_V97D_ and YFP-HINT1_V97D_ to monitor Ap_4_A-induced HINT1 interactions (Fig. 5b). In this experiment, the V97D mutation was introduced to minimize the background FRET signal generated by the classic HINT1 dimerization^48^. The FRET signal between CFP-YFP tandems was measured and set to 100% (Fig. 5b). Only Ap_4_A, but not AMP, Ap_3_A, or Ap_5_A, increased the FRET signal (Fig. 5b), suggesting that ATP, Ap_3_A, and Ap_5_A were not able to induce HINT1 interactions through the Ap_4_A linked interface. Estimated from the FRET signal, 16% of HINT1_V97D_ was recruited into polymer by 700 μM Ap_4_A. Increasing Ap_4_A concentration to 1400 μM further led to 23% of HINT1_V97D_ to polymer. The negative stain EM also showed little effect of Ap_3_A, Ap_5_A, or AMP to induce HINT1 filament compared with Ap_4_A (Fig. 5c–f). Therefore, Ap_3_A and Ap_5_A are not able to polymerize HINT1.

Finally, to reveal the molecular specificity of Ap_4_A to induce HAn, we further solved the co-crystal structures of HINT1_H114A_-ATP and HINT1_H114A_-Ap_3_A (Supplementary Table 1). Only the AMP portion of these molecules were resolved in the adenosine pockets of HINT1, with the remaining atoms of ATP or Ap_3_A untraceable (Fig. 5g, h and Supplementary Table 1). Neither ATP (with only one adenosine moiety) nor Ap_3_A (with a shorter phosphodiester linkage) could recruit HINT1 dimers to form polymer in solution. We also solved the HINT1_H114A_-Ap_5_A structure in a similar crystal packing state as HINT1_H114A_-Ap_4_A. The five-phosphate linkage in Ap_5_A adopted a bent and zigzag conformation compared with the straight conformation of Ap_4_A (Fig. 5i), suggesting that the increased length of Ap_5_A is too long to stabilize the HAn interface as Ap_4_A does. Together, these results indicate that the bivalent characteristic, the length of the phosphodiester linkage, and the interaction of phosphates of Ap_4_A with HINT1 were all critical for HAn formation.

## Discussion

Together, this work shows that the second messenger Ap_4_A directly polymerizes its target protein HINT1. This polymerization interface is also a potential interface for MITF, and therefore oligomerization releases MITF from HINT in solution. In addition, we also observed synchronous changes in HINT1 cellular distribution patterns and the transcription of two MITF downstream genes (*c-Met* and *c-Kit*) during the RBL activation process. These results suggest a mechanism in which Ap_4_A polymerizes HINT1 to block the interaction with MITF, which subsequently releases MITF and activates downstream gene transcription during mast cell activation (Fig. 4i).

Mast cells play critical roles in asthma, allergy, and anaphylaxis, and generate inflammatory reactions by releasing mediators during the immune activation^15^. The aberrant, chronic, or systemic activation of mast cells leads to multiple pathological conditions and promotes harmful inflammation that damages host tissues^50,51^. Therefore, the activation of mast cells upon antigen stimulation must be tightly controlled. This stimulation-induced synthesis of Ap_4_A molecules leads to subsequent binding to HINT1 homodimers, thereby creating a HAn filament that is driven by a unique symmetric adenosine moieties in the second messenger Ap_4_A. This process requires a remarkable structural precision of Ap_4_A, in a length-dependent manner of its tetra-phosphate linkage. HINT1 polymerization may block the MITF-HINT1 interface on HINT1 that releases MITF for the transcriptional activation. Our results on the Ap_4_A-HINT1-MITF pathway at the molecular level provides a basis for developing potential approaches to treating asthma and anaphylaxis.

HINT1 is involved in a broad spectrum of deficiencies in mammals. It is an ancient and conserved haplo-insufficient tumor suppressor. The deficiency of HINT1 in mice results in increased susceptibility to both spontaneous and carcinogen-induced tumor formation^32,33^. The regulation of HINT1-MITF is also found in melanoma cells^52^. MITF is the master regulator of melanocyte development and melanoma formation, as well as proliferation and relapse^53–55^. K21 and Y109 in HINT1, which are located on the HAn interface, are two of the most prevalent posttranslationally modified residues in HINT1 in multiple cancers including colorectal, liver, gastric cancer, and leukemia^49^. We show that the mutants K21D and Y109D lost the ability to polymerize (Supplementary Fig. 9b–e) and are also unable to regulate MITF^49^. Further, loss-of-function mutations in HINT1 were found in inherited peripheral neuropathies^56–59^. Importantly, the tumor suppressor functions of HINT1 appear to be independent of its enzymatic activity, as a mutant HINT1 (H112N) defective in AMP-NH_2_ hydrolyzing activity was not impaired for induction of apoptosis^60^. Here, the finding that Ap_4_A regulates HINT1 through induction of polymerization hints that this non-enzymatic regulation may serve as a broad-spectrum mechanism for HINT1 functions.

It has been widely reported that second messengers regulate target proteins by modifying their structural conformations^40–42,61^. For example, 3′,5′-cyclic AMP binds to the regulatory subunit of protein kinase A (PKA) causing a dramatic conformational change that uncouples the large lobe of the catalytic subunit of PKA^40^; InsP_3_ binds to the ligand-binding core of InsP_3_ receptors and evokes conformational changes that open the channel gate to release Ca^2+^^41^; the c-di-AMP binding induces dramatic changes in the orientation and position of the two CT domains (a rotation of ∼4.5°) of *Listeria monocytogenes* (LmPC)^42^; c-di-GMP binds to the STING dimer and induces a conformational change with 16 Å between the apical monomer wing domains and triggers an ∼26° rotation in monomer wing domains from the apo state^61^, and so on. However, Ap_4_A-complexed HINT1 showed a minimal structural difference to the apo HINT1 structure with RMSD < 0.238 Å. Apparently, the direct effect from polymerization, which blocks the MITF-HINT1-binding site, is probably even more effective and direct than potential conformational changes.

Molecular symmetry is common among second messengers such as c-di-AMP, c-di-GMP, cyclic oligoadenylate family, and the Ap_*n*_A family. Cyclic di-GMP binds to its target, the immunosensor protein STING dimer, in a symmetric form^62^. Cyclic di-GMP also forms a symmetric dimer itself through stacking of the guanines and binds two VpsT proteins into a VpsT dimer to regulate motile to sessile transition in vibrio^63^. Cyclic di-AMP adopts a U-shaped conformation to bind dimer interface of two CT domains of pyruvate carboxylase of LmPC^42^. The tetrameric c-di-GMP mediates effective dimerization of transcription factor BldD to control *Streptomyces* development^64^. Compared with them, Ap_4_A appears to be an outlier, which utilizes its symmetry, but also uses its consecutiveness to tether the target proteins into a polymer.

In summary, this work shows that the second messenger Ap_4_A activates HINT1 by directly linking HINT1 proteins into a HAn polymer, highlighting a polymerization-driven signaling mechanism to propagate the allergic response in the innate immune reaction.

## Methods

### Protein preparation

Human full-length WT or mutant HINT1 protein (residue 1–126) was constructed with an N-terminal 6×His-tag with a TEV protease cleavage site in a pHisTEV vector. HisTEV-HINT1 expression was induced by isopropyl β-d-1-thiogalactopyranoside in the bacterial strain BL21 (DE3) at 16 °C for 20 h and the His-tagged protein was purified to homogeneity with a Ni-HiTrap affinity column. The purified protein was further polished by a size-exclusion column (Superdex 75, GE Healthcare, Piscataway, NJ) for biochemical assays. For crystallization, the 6×His-tag was cleaved by TEV protease before gel filtration.

### Crystallization, data collection, and structure refinement

Crystallization was done by the sitting drop method. To co-crystallize HINT1 with ATP/Ap_3_A/Ap_4_A/Ap_5_A, 30 mg ml^−1^ HINT1 protein (WT, H114A) was mixed with 10 mM different ligands (ATP, Ap_3_A, Ap_4_A, or Ap_5_A) separately and incubated on ice for 0.5 h. A drop was then prepared by mixing 0.15 μl of HINT1-ligand solution with 0.15 μl of precipitant solution, containing 100 mM HEPES pH 7.5, 35–40% PEG3350. Crystals were obtained after incubation at 18 °C for 3–7 days. To obtain the HINT1 Ap_4_A soaked crystals, the HINT1_H114A_-Ap_4_A co-crystals were further soaked in solution containing 100 mM HEPES pH 7.5, 35–40% PEG3350, 20 mM Ap_4_A for 4 h.

The crystals were flash-frozen in liquid nitrogen with cryo-solution containing 0.75 mM HEPES pH 7.5, 26–30% PEG3350, and 25% glycerol for data collection. The datasets were obtained from beam line LS-CAT 21-ID-F at Advanced Photon Source (Argonne, IL) or beam line 7-1 at the Stanford Synchrotron Radiation Laboratory (Menlo Park, CA). All datasets were processed with HKL2000^65^. The structures were solved by molecular replacement using the HINT1 structure (PDB: 4EQE) with the program Molrep^66^. Iterative model building and refinement were performed by using Coot and Phenix^67,68^. Data collection and refinement statistics are given in Supplementary Table 1. Representative views of the electron density maps are shown in Supplementary Fig. 12. In the HINT1_H114A_-Ap_4_A and HINT1_H114A_-Ap_5_A co-crystal structures, the Ap_4_A and Ap_5_A bind two HINT1 dimers in two alternative modes, which are related by a twofold symmetry. For both structures, one of the two equivalent modes are selected for analysis in the manuscript.

### Electrophoretic mobility shift assay and immunoblotting

HINT1_WT_ (25 μM) or HINT1_H114A_ were mixed with either 700 μM Ap_3_A, Ap_4_A, Ap_5_A, AMP, ATP, or blank buffer and incubated at 4 °C rotating gently for 2 h. The working buffer includes 25 mM HEPES pH 8.0, 400 mM NaCl, and 2 mM EDTA. Samples were separated by 4–20% gradient SDS-polyacrylamide gel electrophoresis (PAGE) without pre-heating. RealBand 3-color High Range Protein Marker (BBI Life Science, Rockville, MD) was used to identify molecule weight of target bands. Gel under 25 kDa marker was cut for Coomassie brilliant blue staining. Gel above 25 kDa was transferred onto nitrocellulose membranes. Blottings were blocked in 5% non-fat milk for 30 min at room temperature, then incubated in Anti-His antibody (TransGen Biotech, catalog number HT501-02, dilution 1:3000) overnight at 4 °C and follow by 1 h incubation in horseradish peroxidase (HRP)-conjugated Goat Anti-Mouse IgG (BBI Life Science, catalog number D110087, 1:5000 dilution) at room temperature. Blottings were then treated with Pierce ECL Western Blotting Substrate and visualized with Amersham TM Imager 600 (GE Healthcare, Piscataway, NJ). The molecular weights of protein bands were calculated with three independent experiments by Amersham TM Imager 600 Analysis Software Version1.0 (GE Healthcare, Piscataway, NJ).

### Negative stain electron microscopy

HINT1_WT_ proteins were mixed with either Ap_3_A, Ap_4_A, Ap_5_A, AMP, or blank buffer. The final system contained 2 mM EDTA, 25 mM HEPES pH 8.0, 400 mM NaCl, 10 μM HINT1_WT_ protein, and substrates with indicated concentration (e.g., 700 μM for AMP/Ap_3_A/Ap_4_A/Ap_5_A, 400 μM for Ap_4_A) Samples were well mixed and incubated at 4 °C for 2 h. Five microliters of each sample was then transferred directly to a freshly glow discharged transmission electron microscope (TEM) grid (Beijing Zhongjingkeyi Technology Co.,Ltd) for 45 s. The grid was rinsed with 5 μl 3% w/v uranyl acetate pre-cooled at 4 °C, followed by staining with 5 μl 3% w/v uranyl acetate for 45 s. The excess buffer was removed. The grid was dried in air before TEM imaging. Images of the specimens were obtained at 120 kV on a FEI T12 Tecnai EM.

### Fluorescence anisotropy assay

Fluorescence anisotropy measurements were carried out using a SpectraMax M5 plate reader (Molecular Devices, Sunnyvale, CA). The interaction between 5IAF-labeled MITF (_5IAF-_MITF) and HINT1_WT_ through fluorescence anisotropy assay was first established based on a previous study^49^. HINT1_WT_ (final concentration 500 nM) was mixed with gradient Ap_4_A and ATP (0–1400 μM) in buffer containing 20 mM HEPES pH 7.5, 200 mM NaCl, 1 mM MgCl_2_, 1% (v/v) Glycerol. After a 2 h incubation at 4 °C, _5IAF-_MITF was added to the mixtures to initiate the reaction with final concentration of 150 nM. The measurement was performed after 2 h of incubation. Excitation beam at 494 nm and emission at 518 nm were used to measure the fluorescence anisotropy.

### Fluorescence resonance energy transfer assay

FRET measurements were carried out using a SpectraMax M5 plate reader (Molecular Devices, Sunnyvale, CA). Purified 500 nM CFP-HINT1_H114A___V97D_ and 500 nM YFP-HINT1_H114A_V97D_ were incubated with gradient Ap_3_A/Ap_4_A/Ap_5_A/AMP up to 20 μl reaction volume. The reaction buffer contained 20 mM HEPES pH 7.5, 250 mM NaCl. The mixtures were incubated at 4 °C for 3 h before the measurement. Excitation beam at 436 nm and emission at 480 nm, 525 nm were used to detect the FRET efficiency.

### Cell culture and activation

RBL-2H3 cells were obtained from the cell bank of the Chinese Academy of Science (catalog number TCR7), cultured and maintained at 37 °C in growth medium containing RPMI, 2 mM l-glutamine, 2 mM nonessential amino acids, 100 units ml^−1^ penicillin, 100 μg ml^−1^ streptomycin, and 10% fetal bovine serum. For IgE and antigen activation, RBL-2H3 cells were first treated with mouse-IgE antibody (Sigma-Aldrich, catalog number D8406) at 400 ng ml^−1^ for 2 h at 37 °C. Followed by 3× wash with warm phosphate-buffered saline (PBS) buffer, cells were stimulated with 400 ng ml^−1^ DNP-albumin (Sigma-Aldrich).

### Analyses of the endogenous HINT1 oligomerization by EMSA

To analyze endogenous HINT1 oligomers during RBL activation, the cells with or without activation were collected with ice-cold RIPA buffer containing 50 mM Tris-HCL (pH 7.4), 150 mM NaCl, 1% NP-40, 0.1% SDS, 1× protease inhibitor cocktail B14002 (Bimake, Houston, USA) and 1× phosphatase inhibitor cocktail B15002 (Bimake, Houston, USA). The samples were homogenized by passing through a 21-gauge syringe needle and then separated by 8–16% gradient SDS-PAGE without pre-heating. Immunoblots were blocked in 5% non-fat milk for 30 min at room temperature, then incubated in the Anti-HINT1 antibody (Abcam, catalog number ab124912, 1:3000 dilution) for 48 h at 4 °C and followed by 1 h incubation in the HRP-conjugated Goat Anti-rabbit IgG (BBI Life Science, catalog number D110058, 1:5000 dilution) at room temperature. Blottings were then treated with Cyanine 3 dye from TSA^TM^ Plus Fluorescence systems NEL744 (PerkinElmer, Waltham, MA) for 10 min at room temperature shielded from light. Visualization was performed with Amersham TM Imager 600 (GE Healthcare, Piscataway, NJ) under fluorescence detection mode with stimulation light 520 nm and Cy3 filter.

To analyze the polymerization of Flag_HINT1_WT, Flag_HINT1_Y109D, Flag_HINT1_L53R during the RBL activation, an Anti-FLAG antibody (Proteintech, catalog number 20543-1-AP, 1:3000 dilution) was used and followed by 1 h incubation in HRP-conjugated Goat Anti-Rabbit IgG (BBI Life Science, catalog number D110058, 1:5000 dilution) at room temperature. Anti-β tubulin antibody (Proteintech, catalog number 10068-1-AP, 1:3000 dilution), and HRP-conjugated Goat Anti-mouse IgG (BBI Life Science, catalog number D110087, 1:5000 dilution) were used as primary and secondary antibodies for the Tubulin control in the western blotting. Blottings were then treated with Pierce ECL Western Blotting Substrate and visualized with Amersham TM Imager 600 (GE Healthcare, Piscataway, NJ).

### Cell imaging

Cells were collected at different time points after DNP-albumin treatment by fixing the cells with 100% methanol/acetone at −20 °C. Cells were then incubated in HINT1 polyclonal antibody (Proteintech, catalog number 10717-1-AP, 1:500 dilution) for 2 h at room temperature. After washing with PBS, the samples were incubated with the secondary antibody (Abcam, catalog number ab6939, 1:1000 dilution) for 1 h at room temperature and shielded from light. DAPI (4′,6-diamidino-2-phenylindole) staining was used to detect the nucleus. Protein localization was visualized with a laser scanning microscope (Leica-microsystems) both in the confocal mode and the stimulated emission depletion mode (STED). Negative control experiments were done without primary antibodies.

STED images were obtained with a Leica TCS SP8 STED 3× microscope equipped with a white light pulse laser, STED laser (660 nm), an oil-immersion 100×/numerical aperture 1.4 objective lens (HC PL APO CS2, Leica), a TCS SP8 time-gated system, and a hybrid detector with a high sensitivity. The STED depletion laser was co-aligned with the excitation laser and was used to selectively deactivate the fluorescence surrounding the focal point by stimulated emission, while the remaining fluorescence in the center was then detected. It allows an increased resolution high up to 50 nm by shrinking the point-spread function of the microscope. Images were acquired in both confocal and STED mode with 1024 × 1024 pixel formats. Acquisition parameters such as laser power, image size, pixel dwell time, line average, frame accumulation, pinhole value, and time-gating interval (0.5–6 ns post-pulse time window) were optimized for achieving the best imaging quality. All images were deconvolved by Huygens software (Scientific Volume Imaging) with the Huygens classical 282 maximum likelihood estimation deconvolution algorithm and an automatically generated theoretical Point Spread Function. Images were deconvolved with the deconvolution wizard. Default settings were generally adequate; however, certain cases required signal-to-noise ratios and backgrounds to be determined individually. Before deconvolution, we checked parameter settings in the software and confirmed the restoration of the acquisition parameters. Images were further processed with FIJI software (National Institute of Health).

### Reverse-transcription PCR assay

The complementary DNA (50 ng) was used for quantitative PCR (qPCR) amplification with SYBR green PCR master mix (Vazyme Biotech Co., Ltd). The relative levels of expression of genes were normalized according to those of GAPDH. qPCR data were calculated using the comparative Ct method. All qPCR were performed in triplicate and three independent RNA samples were assayed for each time point. Primers are listed in Supplementary Table 2.

### Hydrolysis activity of HINT1 toward Ap_4_A measurement

HINT_WT_ protein (25 μM) was incubated with 1 mM Ap_4_A in the buffer containing 25 mM HEPES pH 8.0, 400 mM NaCl at 37 °C for multiple time courses (0 min, 15 min, 30 min, 1 h, 2 h, 4 h), with total volume of 50 μl. At the end of each incubation, the sample was heated at 95 °C for 10 min and centrifuged at 13,500 × *g* for 10 min. Supernatant was then diluted into 600 μl buffer containing 25 mM HEPES pH 8.0 and analyzed through source 15Q Column by high-performance liquid chromatography.

### Analytical ultracentrifugation

Sedimentation velocity experiments were performed on Beckman XL-I analytical ultracentrifuge under 42,000 r.p.m. (128,420 × *g* at the center, 142,250 × *g* at bottom) at 20 °C. HINT1_H114A_ protein (50 μM) protein with or without 700 μM Ap_4_A were incubated for 1 h at 4 °C before ultracentrifugation. The working buffer contains 20 mM HEPES pH 8.0, 400 mM NaCl, and 2 mM EDTA. The partial specific volume of different protein samples and the buffer density were calculated using program SEDNTERP (http://www.rasmb.bbri.org/). The final sedimentation velocity data were analyzed and fitted to a continuous sedimentation coefficient distribution model using program SEDFIT^69^.

### Generation of HINT1 knockdown RBL cell lines

The pLV-RNAi lentiviral system (Biosettia, SORT-B19) was used to generate lentiviral particles. The nucleotide sequence for short hairpin RNA (shRNA) constructs are listed in Supplementary Table 3. Of note, four shRNA sequences, shHINT1-1, shHINT1-2, shHINT1-3, and shHINT1-4, were designed against the rat *HINT1* gene (NM_008248.3), whereas Scramble-1 and Scramble-2 were predicted not to target any known genes. Each nucleotide was synthesized and inserted into pLV-RNAi-expressing vector.

For lentiviral production, the recombinant plasmids described above and pHelper plasmids (pVSV-G and pCMVΔ8.92) were co-transfected into HEK293T cells using Lipofectamine 3000 (Invitrogen) according to the manufacturer’s instruction. After 48 h, virus-contained culture medium was collected and filtered with 0.45 μm membrane.

For HINT1 knockdown RBL cell establishment, RBL-2H3 cells were seeded and cultured to reach 50% confluence in six-well plates and transduced with constructed lentiviruses described above. Puromycin (1 μg ml^−1^) was used to select stably transduced cells. The knockdown efficiency was determined with western blotting using Anti-HINT1 antibody (Abcam, catalog number EPR5108, 1:1000 dilution) and HRP-conjugated Goat Anti-Rabbit IgG (BBI Life Science, catalog number D110058, 1:5000 dilution). Anti-β Tubulin antibody (Proteintech, catalog number 10068-1-AP, 1:3000 dilution), and HRP-conjugated Goat Anti-mouse IgG (BBI Life Science, catalog number D110087, 1:5000 dilution) were used as primary and secondary antibodies for the Tubulin control in the western blotting.

### Establishment of RBL cells expressing Flag_HINT1_WT and mutants

Human HINT1_WT, HINT1_Y109D, HINT1_V97D, HINT1_I22R, and HINT1_L53R sequence was inserted into pMSCVzeocin vector. For retroviral production, recombinant plasmid contained human HINT1 sequences and pHelper plasmids (pVSV-G and pCMV-Gag) were co-transfected into HEK293 cells using Lipofectamine 3000 (Invitrogen) according to the manufacturer’s instruction. After 48 h, virus-contained culture medium was collected and filtered with 0.45 μm membrane.

For cell infection, HINT1 knockdown RBL cells were seeded and cultured to reach 50% confluence in six-well plates and transduced with constructed retroviruses (Human HINT1_WT, HINT1_Y109D, HINT1_V97D, HINT1_I22R, and HINT1_L53R). After 24 h infection, the virus contained medium was replaced with complete medium. After 72 h infection, cultural medium was replaced with fresh medium containing zeocin (4 μg ml^−1^) to select for stably transduced cells. The transduced cells were passaged every 3 days with zeocin-contained medium until no live cells in the mock control well. The expression efficiency was then determined with western blotting by Anti-FLAG antibody (Proteintech, catalog number 20543-1-AP, 1:3000 dilution), Anti-HINT1 antibody (Abcam, catalog number EPR5108, 1:1000 dilution), in pair with HRP-conjugated Goat Anti-Rabbit IgG (BBI Life Science, catalog number D110058, 1:5000 dilution), Anti-β Tubulin antibody (Proteintech, catalog number 10068-1-AP, 1:3000 dilution), and HRP-conjugated goat anti-mouse IgG (BBI Life Science, catalog number D110087, 1:5000 dilution) were used as primary and secondary antibodies for the Tubulin control in the western blotting.

### Reporting summary

Further information on research design is available in the Nature Research Reporting Summary linked to this article.

## Supplementary information

Supplementary Information

Reporting Summary

Source Data

## Data Availability

The modeled atomic coordinates have been deposited in PDB with accession codes 6J64 (HINT1_H114A_–Ap_4_A^cocrystallization^) [10.2210/pdb6J64/pdb], 6J65 (HINT1_H114A_–Ap_4_A^soaking^) [10.2210/pdb6J65/pdb], 6J5S (HINT1_H114A_–Ap_5_A) [10.2210/pdb6J5S/pdb], 5ED6 (HINT1_H114A_–ATP) [10.2210/pdb5ED6/pdb], 6J58 (HINT1_WT_–Ap_4_A) [10.2210/pdb6J58/pdb], 5ED3 (HINT1_WT_–Ap_5_A) [10.2210/pdb5ED3/pdb], 6J53 (HINT1_WT_–ATP) [10.2210/pdb6J53/pdb], and 6J5Z (HINT1_H114A_–Ap_3_A) [10.2210/pdb6J5Z/pdb]. The source data underlying Figs. 2a, b, 3c, d, 4a, h and 5a, b, and Supplementary Figs. 1c, 7a, 9c, e and 10a–d are provided as a Source Data file. All other source data are available from the corresponding authors upon reasonable request.
