# Supplementary material for: Second messenger Ap4A polymerizes target protein HINT1 to transduce signals in FcεRI-activated mast cells
Source: Nat Commun. 2019 Oct 11;10:4664. doi: 10.1038/s41467-019-12710-8 (PMC6789022; doi:10.1038/s41467-019-12710-8)
Supplement: Supplementary file 1 — Supplementary Information [file 41467_2019_12710_MOESM1_ESM.pdf]

Supplementary Information

Second Messenger Ap_4A Polymerizes Target Protein HINT1 to Transduce Signals in FcεRI-activated Mast Cells

Yu *et al.*

List of Content

Supplementary Tables 1-3

Supplementary Figures 1-12

Supplementary References

Supplementary Table 1. Data collection and refinement statistics

	$HINT1_{WT}$ – ATP	$HINT1_{WT}$ – Ap_4A	$HINT1_{WT}$ – Ap_5A	$HINT1_{H114A}$ – ATP	$HINT1_{H114A}$ – $Ap_4A^{cocrystallization}$	$HINT1_{H114A}$– $Ap_4A^{soaking}$	$HINT1_{H114A}$ – Ap_5A	$HINT1_{H114A}$ – Ap_3A
**Data collection**								
Space group	*C2*	*C2*	*C2*	*C2*	*C2*	*P1*	*C2*	*P1*
Cell dimensions								
a, *b*, *c* (Å)	78.59, 46.50, 63.92	78.72, 46.42, 64.25	78.45, 46.31, 64.10	78.35, 46.39, 64.08	78.82, 46.41, 64.13	46.30 46.57, 64.25	78.66, 46.48, 64.11	45.42, 45.44, 63.92
α, β, γ (°)	90.00, 94.39, 90.00	90.00, 94.90, 90.00	90.00, 94.92, 90.00	90.00, 94.70, 90.00	90.00, 94.23, 90.00	98.06, 89.82, 118.25	90.00, 94.11, 90.00	86.57, 86.48, 61.35
Resolution (Å)	25.00-1.52 (1.55-1.52)*	25.00-1.52 (1.57-1.52)	25.00-1.31 (1.36-1.31)	50.00-1.52 (1.57-1.52)	50.00-0.95 (0.97-0.95)	50.00-1.42 (1.47-1.42)	50.00-1.02 (1.04-1.02)	50.00-1.30 (1.35-1.30)
R_{sym} or R_{merge} (%)	3.6 (10.8)	4.2(27.7)	4.9(27.7)	5.1(19.5)	5.2(38.7)	6.5(42.7)	4.0 (45.6)	5.0(40.5)
I / s*I*	40.5 (12.5)	41.2(7.1)	34.7(5.3)	30.1(8.5)	30.8(3.5)	20.4(3.2)	28.9 (2.1)	25.4(3.1)
Completeness (%)	99.9(99.9)	99.9(100.0)	99.9(98.9)	100.0(100.0)	95.0(80.5)	89.6(92.7)	98.7 (88.3)	95.4(92.0)
Redundancy	3.5(3.0)	7.4(6.5)	7.3 (5.8)	6.4(5.6)	9.8(5.3)	3.8(3.9)	4.0(2.7)	3.9(3.7)
**Refinement**								
Resolution (Å)	25.00-1.52 (1.57-1.52)	25.00-1.52 (1.57-1.52)	25.00-1.31 (1.74-1.31)	50.00-1.52 (1.57-1.52)	50.00-0.95 (0.98-0.95)	50.00-1.42 (1.47-1.42)	50.00-1.02 (1.06-1.02)	50.00-1.30 (1.35-1.30)
No. reflections	35501	35068	51351	34861	137323	78869	115552	94352
R_{work} / R_{free} (%)	15.1/16.9	15.7/17.0	14.4/17.3	14.3/16.4	16.5/17.5	18.0/20.6	16.2/17.5	15.5/17.0
No. atoms								
Protein	1768	1811	1772	1762	1887	3516	1947	3574
Ligand	23	23	23	23	53	53	57	38
Solvent	361	288	282	309	516	791	443	757
B-factors ($Å^2$)								
Protein	11.63	15.20	16.22	14.95	14.33	14.61	11.58	18.02
Ligand	10.58	14.56	15.26	19.17	23.43	17.19	17.13	34.13
Solvent	28.61	32.44	31.84	29.81	30.28	29.18	25.49	31.43
R.m.s. deviations								
Bond lengths (Å)	0.008	0.005	0.009	0.006	0.006	0.009	0.011	0.013

Bond angles (°)	1.109	1.107	1.250	1.192	0.737	1.188	1.342	1.344
Ramachandran plot								
Most favored [%]	100	99	98	99	99	99	99	99
Additional allowed [%]	0	1	2	1	1	1	1	1

*Values in parentheses are for highest-resolution shell.

Supplementary Table 2. Primers for RT-PCR

Name	Sequence (from 5' to 3')
c-Kit forward	GCCCGAAACAAGTCATCTCC
c-Kit reverse	AATTGAAGTCGCCCTGATGC
c-Met forward	TGACGAGTACCGGACGGAGT
c-Met reverse	CGAGAGAGCACCACCTGCAT
GAPDH forward	TCCTGCACCACCAACTGCTTAG
GAPDH reverse	AGTGGCAGTGATGGCATGGACT

Supplementary Table 3. The nucleotide sequences for shRNA constructs

Name	Sequence (from 5' to 3')
shHINT1-1	GTATCCTTCTCTAGGTAGTTTGGATCCAAACTACCTAGAGAAGGATAC
shHINT1-2	GCAAGATCATCCGCAAAGAAATTGGATCCAATTTCTTTGCGGATGATCTTGC
shHINT1-3	GATGATGATGAAAGTCTTCTATTGGATCCAATAGAAGACTTTCATCATCATC
shHINT1-4	GCAGTCTGTCTATCATATTCATTGGATCCAATGAATATGATAGACAGACTGC
Scramble-1	GGATGTGACCTAATGATTTAATTGGATCCAATTAAATCATTAGGTCACATCC
Scramble-2	GTCATTTCCGATAATCCTAGTTTGGATCCAAACTAGGATTATCGGAAATGAC

Supplementary Figures

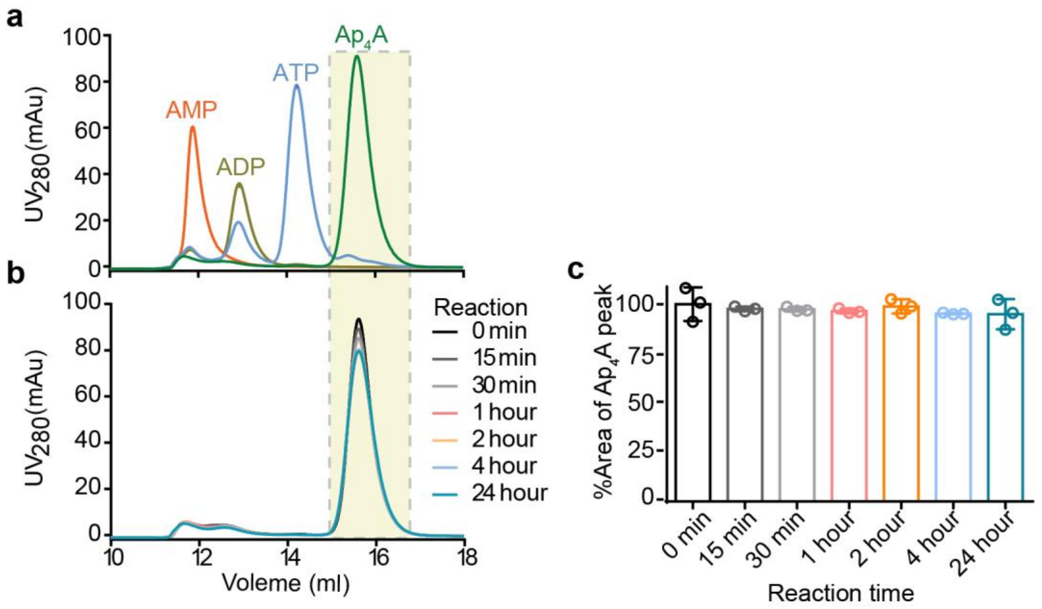

Supplementary Figure 1 | Hydrolysis activity of HINT1$_{WT}$ toward Ap$_4$A. (a) Standard curve of AMP, ADP, ATP, and Ap$_4$A through Source 15Q column by HPLC (High-performance liquid chromatography). (**b)** HINT1$_{WT}$ was incubated with Ap$_4$A at 37 °C for different time points (0 min, 15 min, 30 min, 1 h, 2 h, 4 h, and 24 h). Remaining Ap$_4$A/ATP/ADP/AMP were detected through Source 15Q column by HPLC. (**c**) Relative area of the Ap$_4$A peak following the incubation of HINT1 protein. Ap$_4$A showed minor decreases (< 5 %) after 24 hours reaction with HINT1. Error bars represent the SD of three experimental repeats.

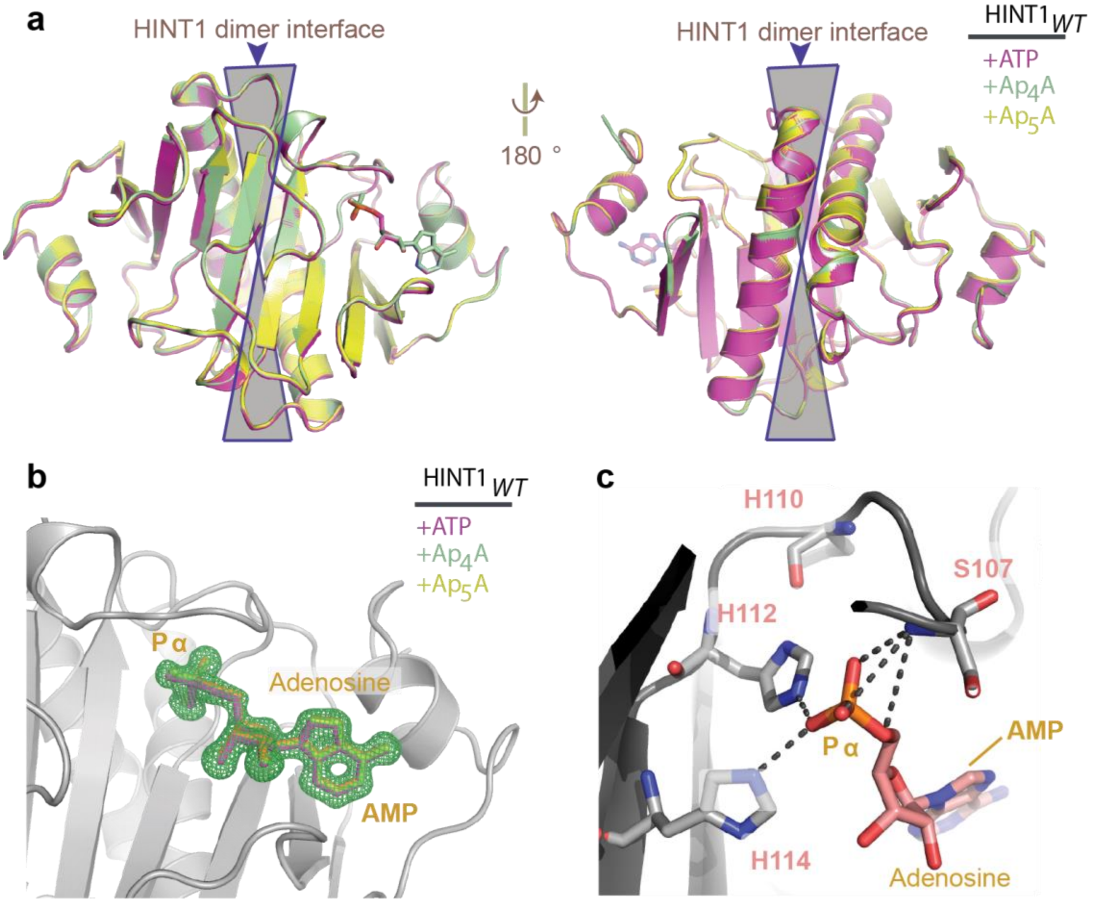

Supplementary Figure 2 | Crystal structures of HINT1$_{WT}$–ATP/Ap$_4$A/Ap$_5$A complex. **(a)** Superimposition of HINT1$_{WT}$ –ATP, HINT1$_{WT}$ –Ap$_4$A and HINT1$_{WT}$ –Ap$_5$A structures with root mean square deviation (RMSD) less than 0.083 Å. Two HINT1 molecules form a dimer through a 5-stranded β-sheet and a central helix. The dimer interface was highlighted in the structure. **(b)** Only AMP was observed in the adenosine-binding pockets of the HINT1$_{WT}$ –ATP, HINT1$_{WT}$ –Ap$_4$A and HINT1$_{WT}$ –Ap$_5$A structures. The representative electron density omit map (Fo-Fc) for AMP was contoured at σ=3.0. **(c)** Recognition of the alpha-phosphate of ATP/Ap$_n$A by HINT1$_{WT}$. The Alpha-phosphate of ATP/Ap$_4$A/Ap$_5$A interacts with H112, H114, and S107. The other phosphates and the 2nd adenosines (for Ap$_4$A/Ap$_5$A) were undetectable in these structures.

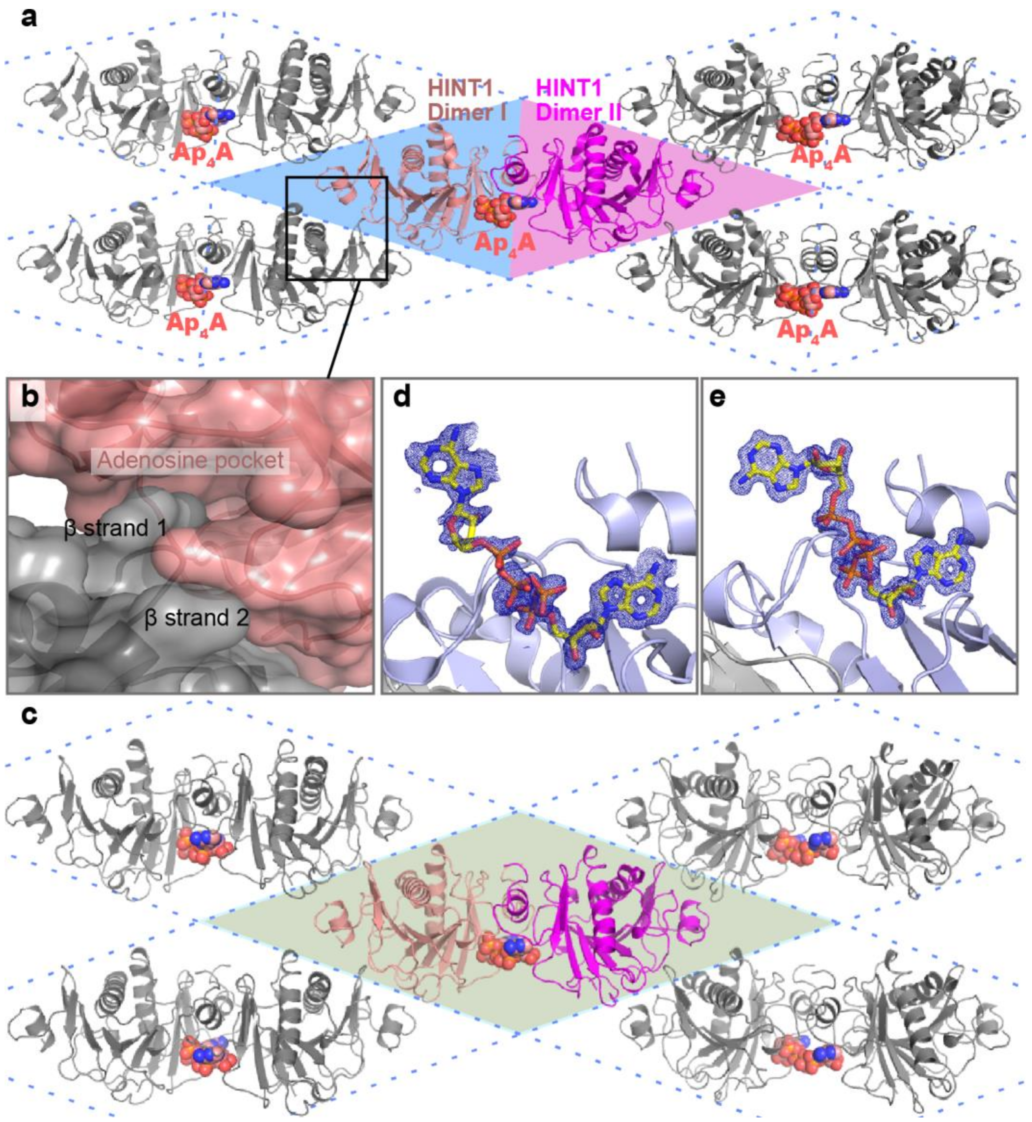

Supplementary Figure 3 | Crystal structures of the HINT1$_{H114A}$-Ap$_4$A Complex. (a) Crystal packing of the HINT1-Ap$_4$A complex structure in HINT1$_{H114A}$-Ap$_4$A^{cocrystallization}. HINT1 forms a dimer and contains two adenosine pockets. In the HINT1-Ap$_4$A complex structure, one adenosine-binding pocket of HINT1 dimer was occupied by Ap$_4$A. **(b)** The other adenosine-binding pocket was blocked by the loop region between β-strand 1 and β-strand 2 from another HINT1 dimer for crystal packing. **(c)** Crystal packing of HINT1-Ap$_4$A complex structure in HINT1$_{H114A}$-Ap$_4$A^{soaking}. Soaking **o**f Ap$_4$A slightly altered the HINT1 tetramer interface, but the overall packing is similar as that of the HINT1$_{H114A}$-Ap$_4$A^{cocrystallization}**. (d)** The electronic density map (2Fo-Fc) for Ap$_4$A in HINT1$_{H114A}$-Ap$_4$A^{cocrystallization} structure was contoured at σ=0.6. **(e)** The electron density map (2Fo-Fc) for Ap$_4$A in the HINT1$_{H114A}$-Ap$_4$A^{soaking} structure was contoured at σ=1.0. Increased Ap$_4$A concentration and shorter incubation time greatly improved the electron density for Ap$_4$A in the soaking structure.

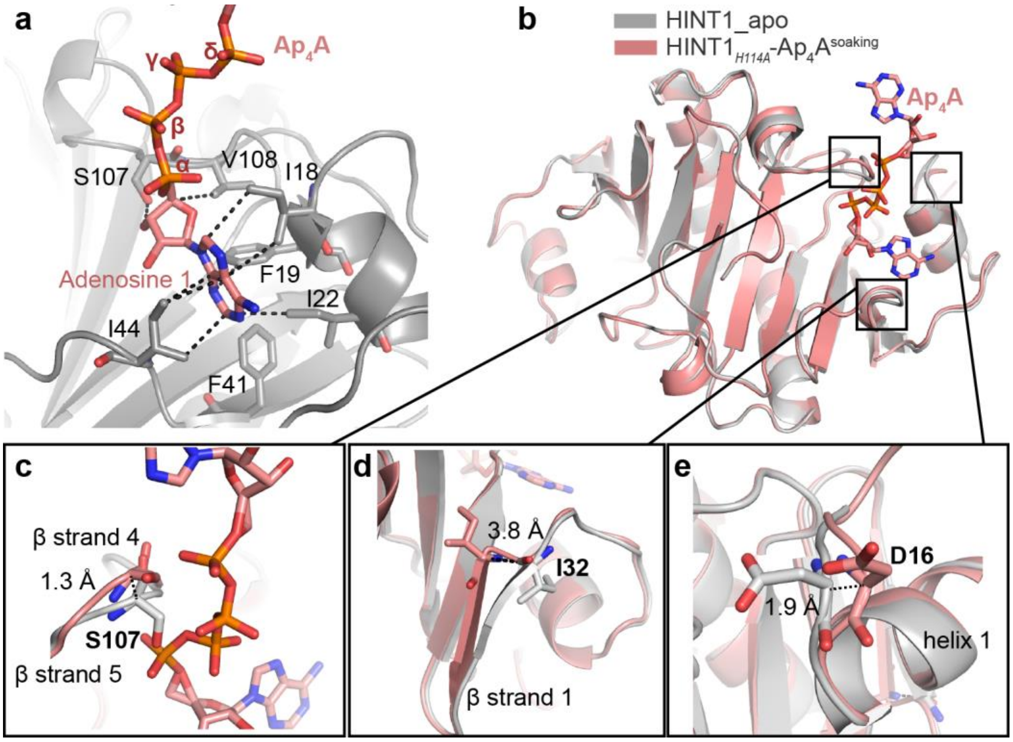

Supplementary Figure 4 | Recognition of Ap_4A by the $HINT1_{H114A}$. (a) Recognition of the adenosine moiety of Ap_4A in the $HINT1_{H114A}$-$Ap_4A^{soaking}$ complex. Six residues (including I18, F19, I22, F41, I44 and V108) of HINT1 formed a hydrophobic adenosine-binding pocket for the first adenosine (A1) of Ap_4A. The remaining atoms of Ap_4A stick out of the pocket of HINT1. **(b)** Ap_4A did not cause significant changes in the HINT1 dimer structure. Superimposition of $HINT1_{H114A}$-$Ap_4A^{soaking}$ structure with HINT1_apo structure (PDB: 1KPB, grey) with root mean square deviation (RMSD) less than 0.238 Å. The only three subtle differences were shown in **(c-e)**.

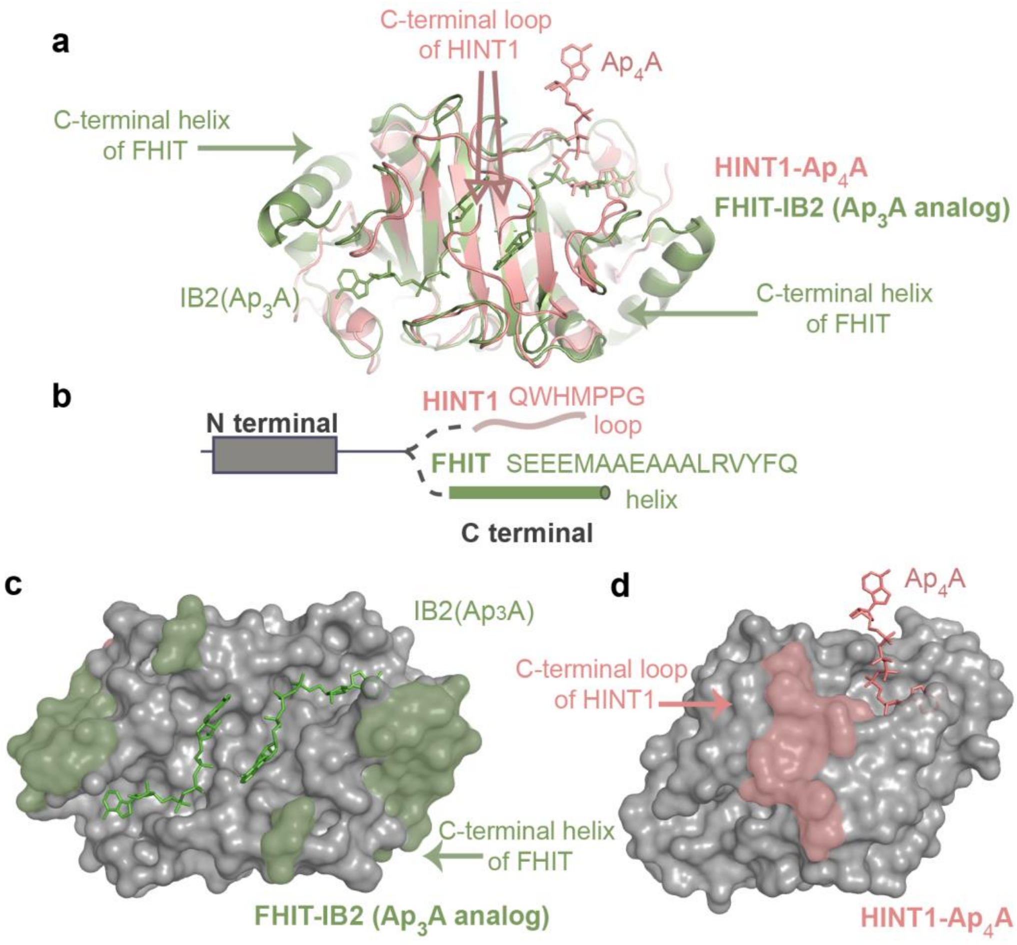

Supplementary Figure 5 | Sticking-out conformation of Ap4A in HINT1-Ap4A complex structure. (a) Alignment of HINT1-Ap4A complex (pink) with FHIT-IB2 structure (green, IB2: Ado-p-CH2-p-ps-Ado, a non-hydrolysable Ap3A analog; PDB: 1FHI). FHIT contains a conserved histidine triad (His-x-His-x-His) sequence motif, and belongs to histidine triad protein (HIT) superfamily together with HINT1[1]. FHIT and HINT1 share a similar tertiary structure and a conserved adenosine-binding pocket, but have different substrate specificities [1]. The proteins of the HINT1 branch in the HIT superfamily hydrolyze adenosine 5′-monophosphoramidate (AMP-NH_2) and ADP, while the proteins of the FHIT branch prefer to hydrolyze di-adenosine polyphosphate (including Ap3A, Ap4A, Ap5A) and ATP[1]. Comparison of the HINT1-Ap4A structure with the FHIT-IB2 structure showed that two IB2 (Ap3A analogs, green) sit in the surface groove formed by two connected adenosine-binding pockets of FHIT dimer [2], while only one Ap4A (pink) was observed in the HINT1-Ap4A complex structure and adopted a unique sticking-out conformation. **(b)** Distinct C-termini between HINT1 and FHIT. FHIT contains a C-terminal helix, while HINT1 contains a conserved and tightly registered C-terminal end loop. **(c)** C-terminal helices of FHIT dimer located on the sides of the adenosine-binding groove and exposed the long-extended ligand groove that run through the FHIT dimer, allowing FHIT to bind two Ap3A analogs side by side in the same groove. While the first adenosine was held in the adenosine-binding pocket

in each subunit, the second adenosines of the two Ap_3A analogs stacked alongside each other and sit flat in the joint ligand groove of FHIT dimer. **(d)** C-terminal loop of HINT1 intersected the ligand groove thus cutting it into two separate small adenosine-binding pockets (20 Å), and each was only enough for one AMP. Ap_4A adopted the sticking-out conformation to fit in the small pocket.

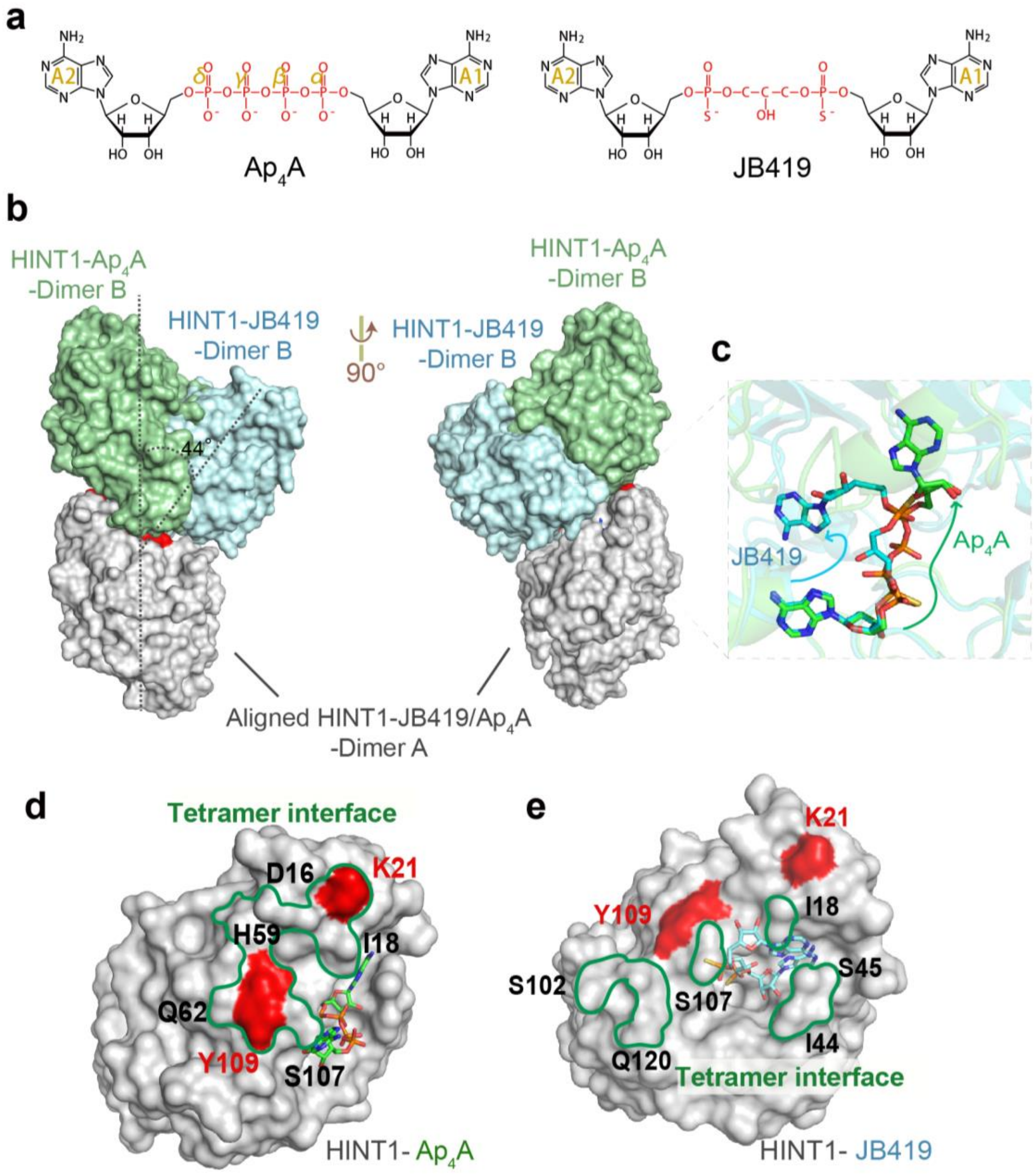

Supplementary Figure 6 | Ap$_4$A and JB419 tether HINT1 dimers differently. (a) Schematics of adenosine tetraphosphates (Ap$_4$A) and the analog JB419. JB419 shares the two adenosine moieties and the length of tetra-phosphate linkage with Ap$_4$A, while the C-C linker of JB419 has less conformational hindrance and charges than the four-phosphate linker of Ap$_4$A. **(b)** Alignment of HINT1-Ap$_4$A complex with HINT1-JB419 structure (PDB: 4ZKL). The adenosine moieties of both JB419 and Ap$_4$A fit into the adenosine binding pockets of HINT1 and thus tether two HINT1 dimers together. Aligning the HINT1-JB419 structure with HINT1-Ap$_4$A shows that Ap$_4$A linked HINT1 is straight, while JB419 linked HINT1 is bent (rotated ~ 44.4°). **(c)** 4-phosphate linker of Ap$_4$A was less extended than the C-C linker of JB419 in complex with HINT1. **(d-e)** JB419 and Ap$_4$A induced the distinct tetramer interface of HINT1. The tetramer interfaces of two different HINT1 complexes are circled in green.

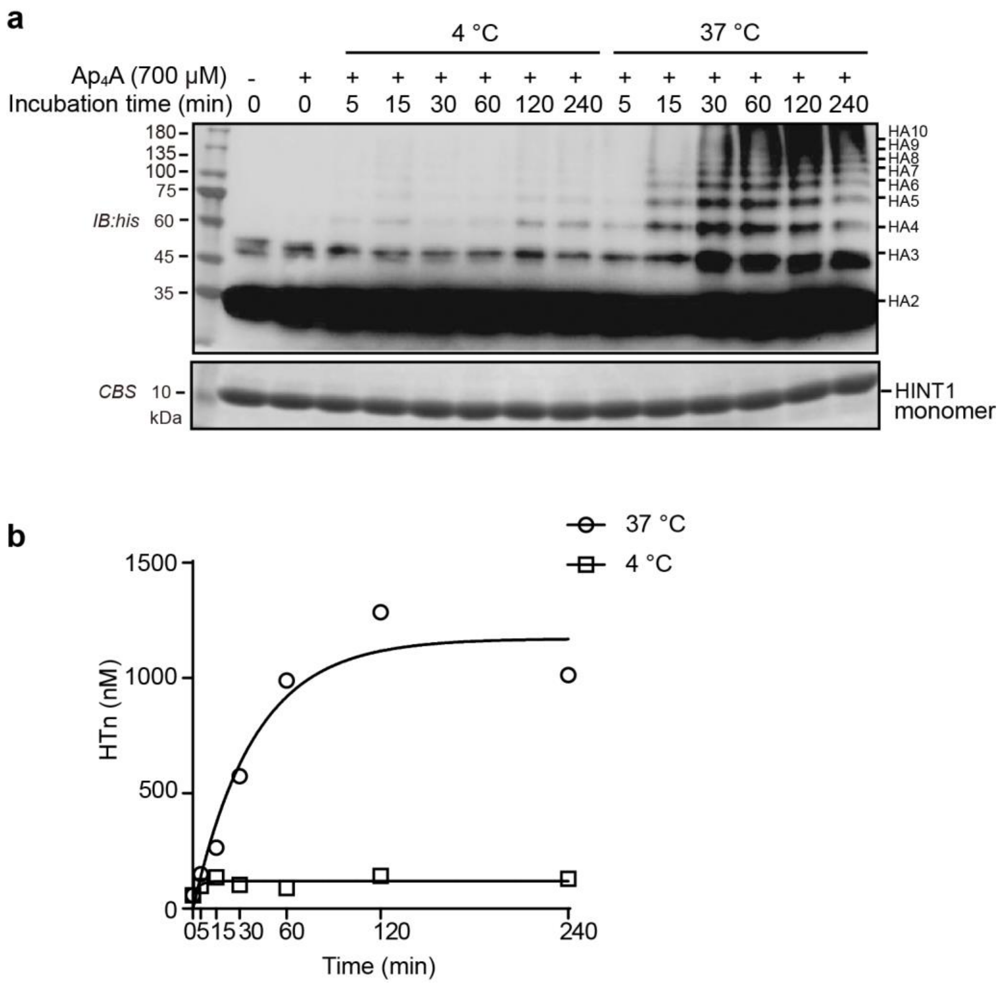

Supplementary Figure 7 | HINT1 polymerization *in vitro*. (a) 25 μM $HINT1_{WT}$ was incubated with Ap_4A for 0 min, 15 min, 30 min, 1 h, 2 h, 4 h separately, at two temperatures (4 °C and physiological 37 °C). The resulting HINT1 protein was loaded onto the gel and monitored through EMSA assay. **(b)** Ratios of HINT1 dimer and polymers in the EMSA assay were analyzed in the Amersham Imager 600 Analysis Software. The polymerization kinetics of $HINT1_{WT}$ were fitted with the equation "$y=a\times(1-\exp(k_{poly}\times t/a))+y_0$", in which the k_{poly} represented the polymerization rate at t=0. Ap_4A drives HINT1 polymerization with an estimated polymerization rate (k_{poly}) of 0.5 $nM\cdot s^{-1}$ at 37 °C.

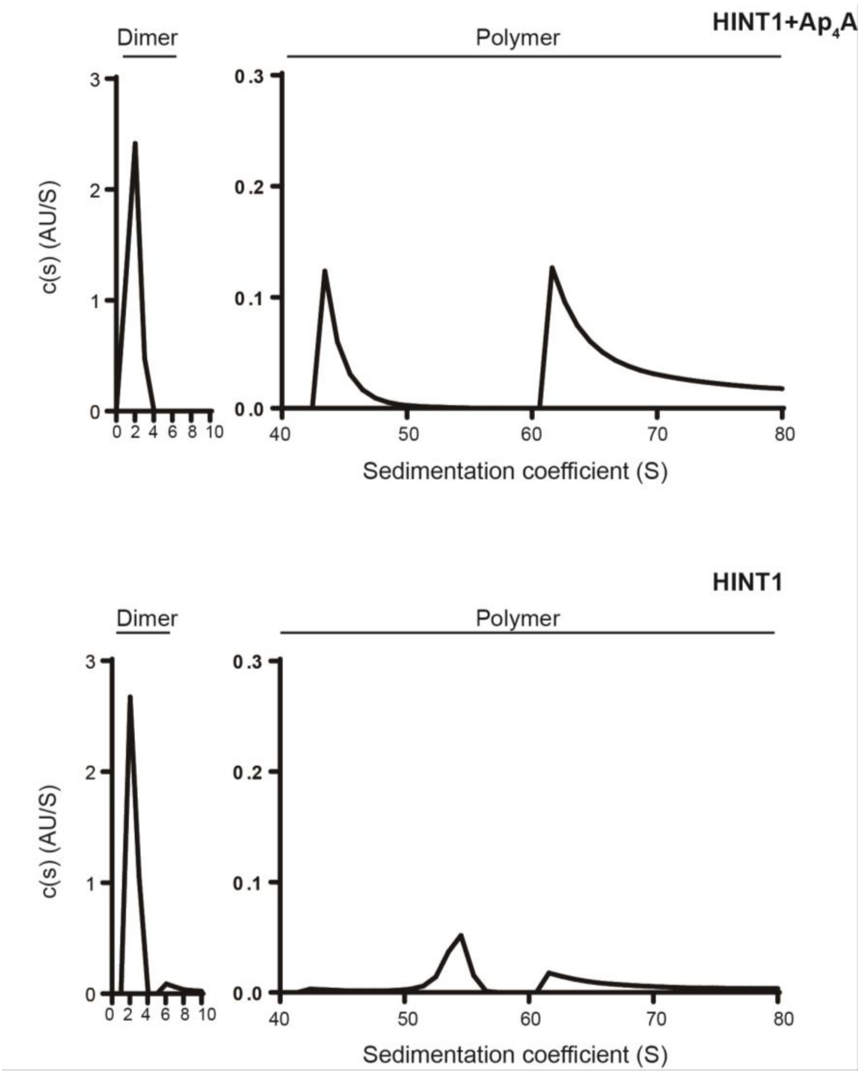

Supplementary Figure 8 | Sedimentation velocity analysis of Ap_4A-induced HINT1 polymerization *in vitro*. Ap_4A induced the formation of high order HINT1-Ap_4A polymers with sedimentation coefficient (S) at 45S (approximately 2.2 MDa) and 62S (approximately 4.2 MDa). HINT1 alone forms limited self-oligomerization. About 15% of HINT1 formed high-order polymer after Ap_4A treatment in this assay.

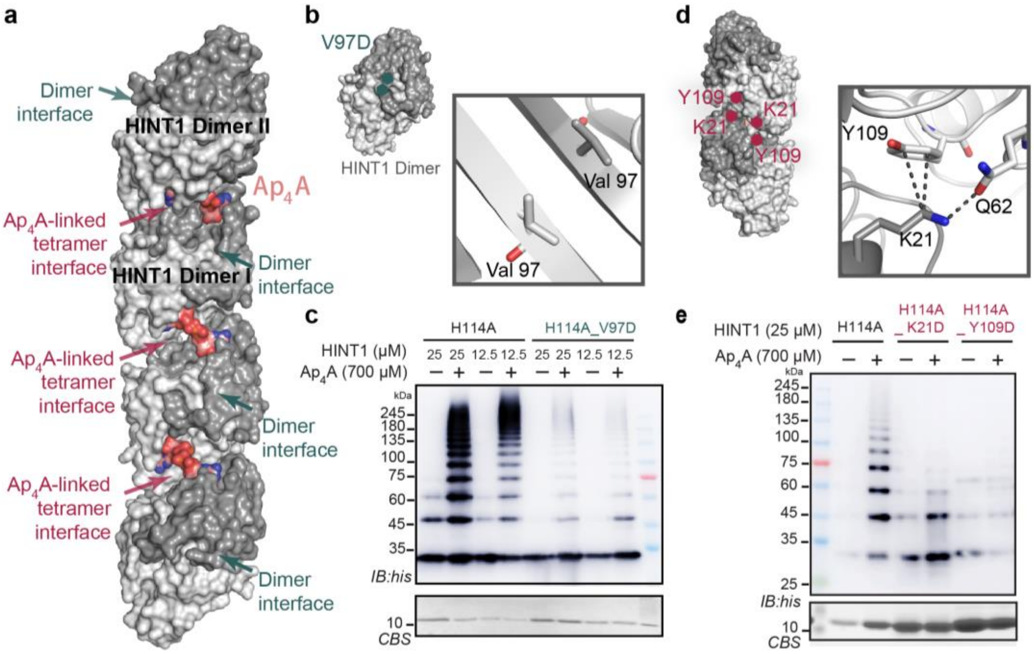

Supplementary Figure 9 | Both the canonical HINT1 dimer interface and Ap$_4$A-linked interface are essential for HAn formation. (a) Model of a HAn polymer. Two interfaces contributed to the HAn formation: one is the canonical HINT1 dimer interface (highlighted with green arrow), and the other is Ap$_4$A-linked tetramer interface (highlighted with red arrow). **(b-c)** Electrophoretic mobility shift assay (EMSA) shows that HINT1 dimer-deficient mutation V97D [3] reduced the ability to form HAn polymer. **(d-e)** EMSA shows that mutations on the Ap$_4$A-linked tetramer interface – K21D and Y109D attenuated the HAn formation.

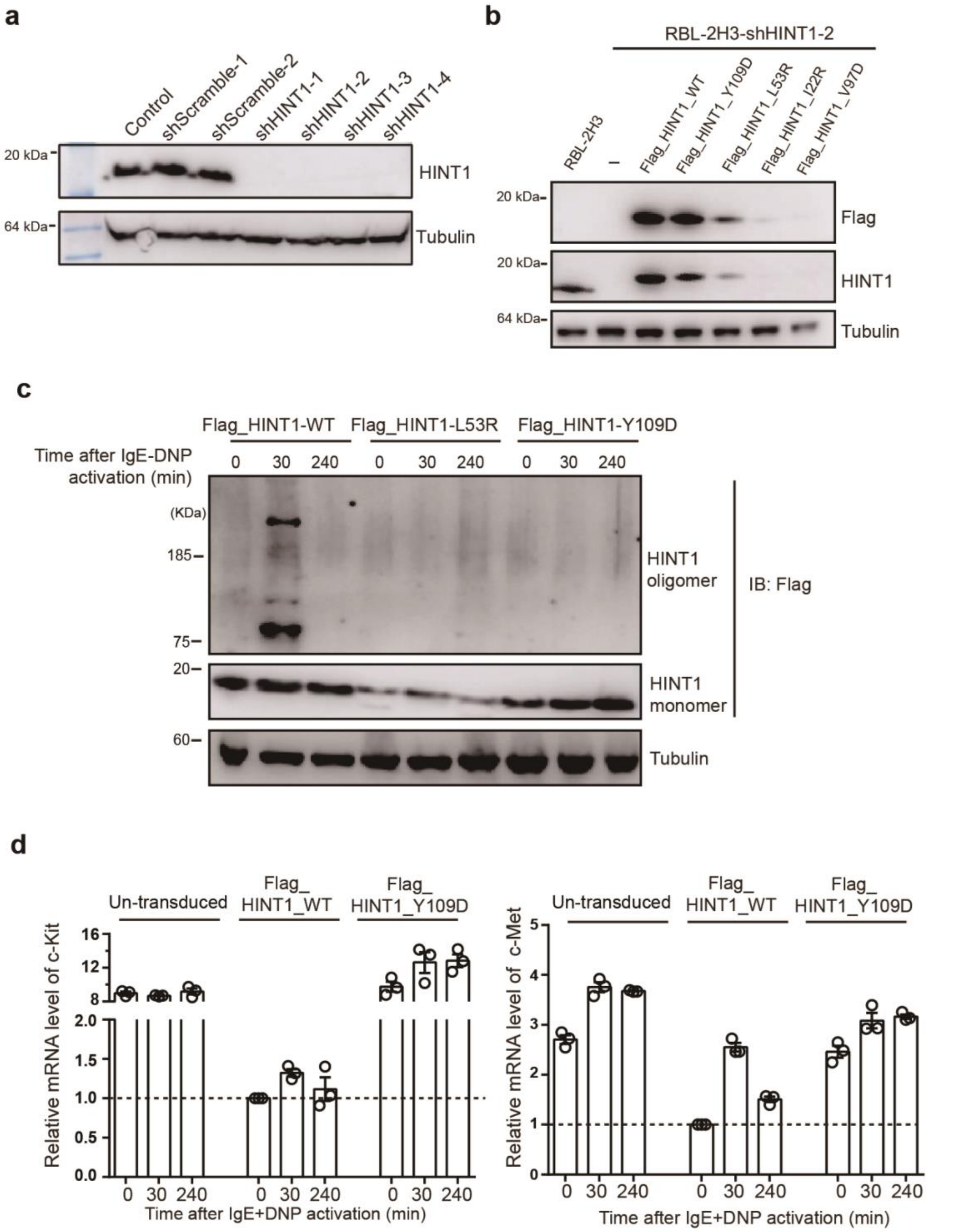

Supplementary Figure 10 | MITF transcriptional activity in RBL cells that stably expressing HINT1 WT and mutant. (a) Western blot result showing the establishment of HINT1 knockdown RBL cells. Four different shRNA's (shHINT1-1/2/3/4) were designed and tested to knock down the expression of HINT1, with two scramble controls (shScramble-1/2). **(b)** Western blots of overexpressed Flag_HINT1_WT or mutants including Y109D, L53R, I22R, and V97D in HINT1 knock-down RBL stable cells (RBL-2H3-shHINT1-2). Those mutations were designed based on the structural information to disrupt the Ap_4A induced HINT1 polymerization

(Y109D located at the tetramer interface; L53R and I22R located at the adenosine binding pocket; V97D located at the dimer interface). Only Flag_HINT1_Y109D normally expressed as WT, while the rest mutants yield greatly reduced protein expressing level. **(c)** Western blots of overexpressed Flag_HINT1_WT, Flag_HINT1_L53R, and Flag_HINT1_Y109D following by the IgE and antigen stimulation in HINT1 knock-down RBL cells expressing Flag_HINT1_WT, Flag_HINT1_L53R, and Flag_HINT1_Y109D. 0 indicates the unstimulated state. Source data are provided as a Source Data file. **(d)** The transcript level of c-Kit and c-Met following the IgE and antigen stimulation in HINT1 knock-down RBL stable cells (labelled as un-transduced), HINT1 knock-down RBL cells stably expressing Flag_HINT1_WT and Y109D. HINT1 knock-down RBL cells are from RBL-2H3-shHINT1-2 as mentioned in **(a)**. Error bars represent the SEM of three experimental repeats.

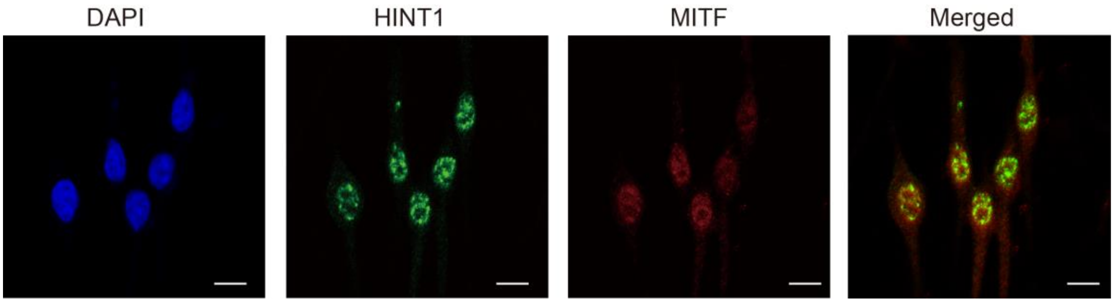

Supplementary Figure 11 | Confocal microscopy image of RBL cells in quiescent state. Localization of HINT1 (green) and MITF (red) in quiescent/un-activated RBL cells is shown through confocal microscopy. Nuclei were labeled with DAPI (blue). Scale bars, 10 um. MITF showed a patterned distribution in nucleus and co-localized with the HINT1 dots. One representative experiment out of three is shown.

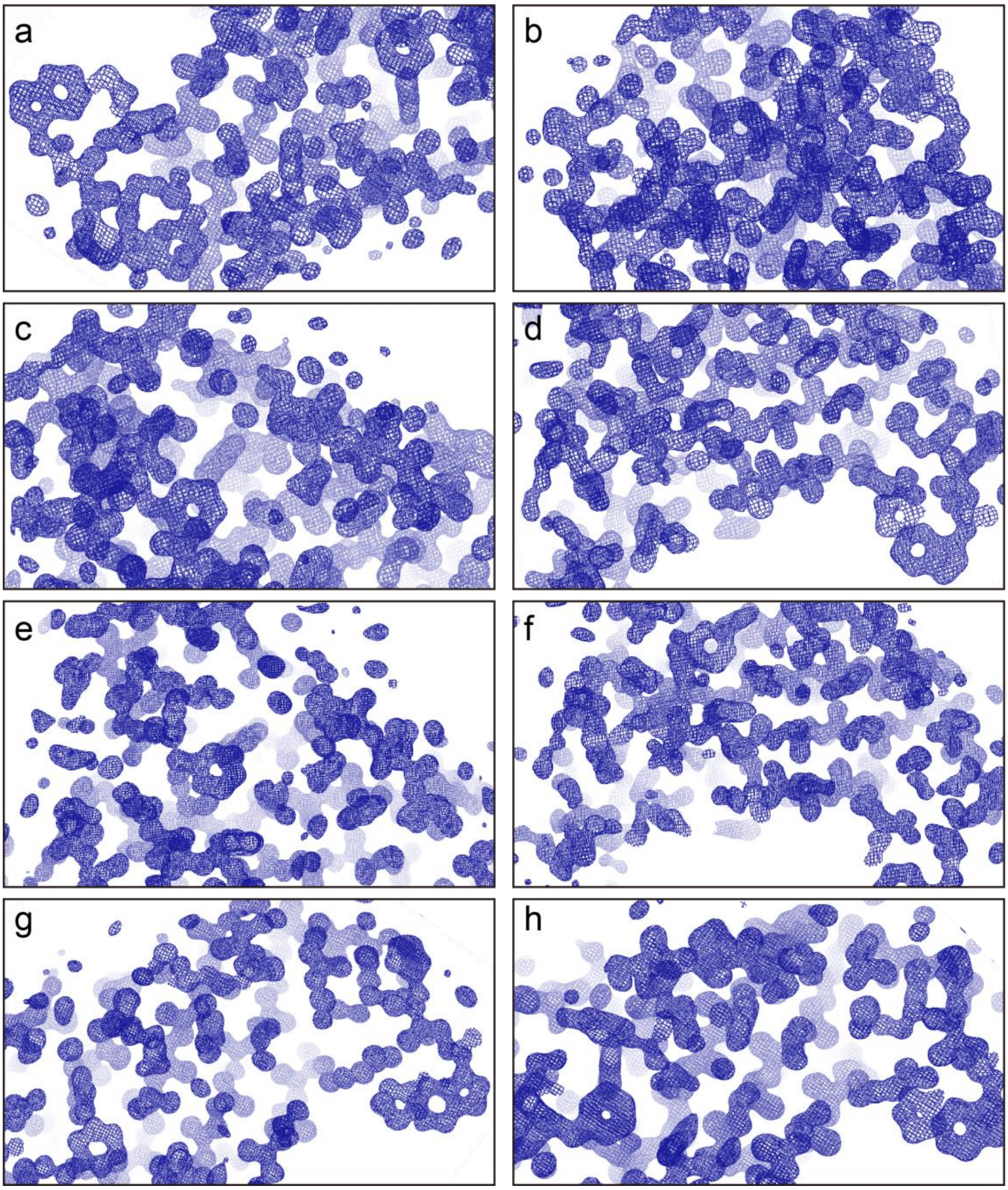

Supplementary Figure 12 | Representative views of the electron density map for HINT1 complex structures. (a) HINT1$_{WT}$–ATP. **(b)** HINT1$_{WT}$–Ap$_4$A. **(c)** HINT1$_{WT}$–Ap$_5$A.**(d)** HINT1$_{H114A}$– ATP. **(e)** HINT1$_{H114A}$–Ap$_4$A^{cocrystallization}. **(f)** HINT1$_{H114A}$–Ap$_4$A^{soaking}. **(g)** HINT1$_{H114A}$–Ap$_5$A. **(h)** HINT1$_{H114A}$–Ap$_3$A. For **a-h**, 2Fo-Fc maps are contoured at σ=1.5.

Supplementary References:

1. Lima CD, Klein MG, Hendrickson WA. Structure-based analysis of catalysis and substrate definition in the HIT protein family. *Science* **278**, 286-290 (1997).
2. Pace HC, Garrison PN, Robinson AK, Barnes LD, Draganescu A, Rosler A, *et al.* Genetic, biochemical, and crystallographic characterization of Fhit-substrate complexes as the active signaling form of Fhit. *Proc Natl Acad Sci U S A* **95**, 5484-5489 (1998).
3. Chou TF, Tikh IB, Horta BA, Ghosh B, De Alencastro RB, Wagner CR. Engineered monomeric human histidine triad nucleotide-binding protein 1 hydrolyzes fluorogenic acyl-adenylate and lysyl-tRNA synthetase-generated lysyl-adenylate. *The Journal of biological chemistry* **282**, 15137-15147 (2007).
