# Supplementary material for: Second messenger Ap4A polymerizes target protein HINT1 to transduce signals in FcεRI-activated mast cells
Source: Nat Commun. 2019 Oct 11;10:4664. doi: 10.1038/s41467-019-12710-8 (PMC6789022; doi:10.1038/s41467-019-12710-8)
Supplement: Supplementary file 2 — Reporting Summary [file 41467_2019_12710_MOESM2_ESM.pdf]

nature research

Corresponding author(s): Jing Wang

Last updated by author(s): Sep 20, 2019

Reporting Summary

Nature Research wishes to improve the reproducibility of the work that we publish. This form provides structure for consistency and transparency in reporting. For further information on Nature Research policies, see Authors & Referees and the Editorial Policy Checklist.

Statistics

For all statistical analyses, confirm that the following items are present in the figure legend, table legend, main text, or Methods section.

n/a	Confirmed	
☐	☒	The exact sample size (n) for each experimental group/condition, given as a discrete number and unit of measurement
☐	☒	A statement on whether measurements were taken from distinct samples or whether the same sample was measured repeatedly
☐	☒	The statistical test(s) used AND whether they are one- or two-sided<br>*Only common tests should be described solely by name; describe more complex techniques in the Methods section.*
☒	☐	A description of all covariates tested
☐	☒	A description of any assumptions or corrections, such as tests of normality and adjustment for multiple comparisons
☐	☒	A full description of the statistical parameters including central tendency (e.g. means) or other basic estimates (e.g. regression coefficient) AND variation (e.g. standard deviation) or associated estimates of uncertainty (e.g. confidence intervals)
☐	☒	For null hypothesis testing, the test statistic (e.g. F, t, r) with confidence intervals, effect sizes, degrees of freedom and P value noted<br>*Give P values as exact values whenever suitable.*
☒	☐	For Bayesian analysis, information on the choice of priors and Markov chain Monte Carlo settings
☒	☐	For hierarchical and complex designs, identification of the appropriate level for tests and full reporting of outcomes
☒	☐	Estimates of effect sizes (e.g. Cohen's d, Pearson's r), indicating how they were calculated

Our web collection on statistics for biologists contains articles on many of the points above.

Software and code

Policy information about availability of computer code

Data collection	See the methods section for details. Soft Max Pro 7.0, Leica Application Suite X, TEM Imaging Analysis software, BM eagle CCD camera, QuantStudioTM Design&Analysis Desktop Software.
Data analysis	See the methods section for details. Microsoft Excel, Graphpad Prism, Amersham TM 21 Imager 600 ANALYSIS SOFTWARE Version1.0, QuantStudioTM Design&Analysis Desktop Software, Leica Application Suite X, TEM Imaging Analysis software, Coot, Phenix, Molrep.

For manuscripts utilizing custom algorithms or software that are central to the research but not yet described in published literature, software must be made available to editors/reviewers. We strongly encourage code deposition in a community repository (e.g. GitHub). See the Nature Research guidelines for submitting code & software for further information.

Data

Policy information about availability of data

All manuscripts must include a data availability statement. This statement should provide the following information, where applicable:

- Accession codes, unique identifiers, or web links for publicly available datasets
- A list of figures that have associated raw data
- A description of any restrictions on data availability

The modeled atomic coordinates have been deposited in PDB with accession codes 6J64 (HINT1H114A–Ap4Acocrystallization), 6J65 (HINT1H114A–Ap4Asoaking), 6J5S (HINT1H114A– Ap5A), 5ED6 (HINT1H114A–ATP), 6J58 (HINT1WT –Ap4A), 5ED3 (HINT1WT –Ap5A), 6J53 (HINT1WT –ATP) and 6J5Z (HINT1H114A–Ap3A). All other source data are available from the corresponding authors upon reasonable request.

Field-specific reporting

Please select the one below that is the best fit for your research. If you are not sure, read the appropriate sections before making your selection.

☒ Life sciences ☐ Behavioural & social sciences ☐ Ecological, evolutionary & environmental sciences

For a reference copy of the document with all sections, see nature.com/documents/nr-reporting-summary-flat.pdf

Life sciences study design

All studies must disclose on these points even when the disclosure is negative.

Sample size	Standard practices for crystallographic, biochemical and cell biological experiments were followed.
Data exclusions	Data exclusion in crystallographic datasets (outlier reflection rejection) was carried out automatically as implemented in the program aimless using pre-established criteria.
Replication	All experimental findings were confirmed in at least two, usually three to five independent experiments. See the figure captions for more details.
Randomization	The samples were allocated into experimental groups randomly.
Blinding	The investigators were not blinded during data collection and analysis. The experiments were checked and confirmed by at least two investigators.

Reporting for specific materials, systems and methods

We require information from authors about some types of materials, experimental systems and methods used in many studies. Here, indicate whether each material, system or method listed is relevant to your study. If you are not sure if a list item applies to your research, read the appropriate section before selecting a response.

Materials & experimental systems

n/a	Involved in the study	
☐	☒	Antibodies
☐	☒	Eukaryotic cell lines
☒	☐	Palaeontology
☒	☐	Animals and other organisms
☒	☐	Human research participants
☒	☐	Clinical data

Methods

n/a	Involved in the study	
☒	☐	ChIP-seq
☒	☐	Flow cytometry
☒	☐	MRI-based neuroimaging

Antibodies

Antibodies used	Anti-His antibody (TransGen Biotech, catalog no. HT501-02), HRP-conjugated Goat Anti-Mouse IgG (BBI Life Science, catalog no. D110087), Anti-HINT1 antibody (Abcam, catalog no. ab124912), HRP-conjugated Goat Anti-rabbit IgG (BBI Life Science, catalog no. D110058), Anti-FLAG antibody (Proteintech, catalog no. 20543-1-AP), Anti-Beta Tubulin antibody (Proteintech, catalog no. 10068-1-AP). Details were included in the Methods.
Validation	All antibodies were validated by the supplier, and were checked in the lab by Western Blotting on cell lysate and by comparing to the manufacturer's results.

Eukaryotic cell lines

Policy information about cell lines

Cell line source(s)	RBL-2H3 cells were kindly provided by Stem Cell Bank, Chinese Academy of Sciences (#TCR 7)
Authentication	The cell line has not been authenticated. Cells displayed homogeneous, clear RBL-like morphology and de-granulation upon IgE-Antigen as expected.
Mycoplasma contamination	The cell line was tested negative for mycoplasma contamination.
Commonly misidentified lines (See ICLAC register)	No commonly misidentified cells were used
